# Pan-genomic dissection of the *CCT* gene family in wheat reveals evolutionary dynamics and regulatory divergence

**DOI:** 10.3389/fpls.2026.1978297

**Published:** 2026-10-26

**Authors:** Wenjie Zheng, Peng Li, Baiqiang Yan, Miao Wang, Xin Sun, Ying Liu, Wei Li, Chen Huang, Mingzhu Sun, Zhengyong Cui

**Affiliations:** 1 Crop Research Institute, Shandong Academy of Agricultural Sciences/National Engineering Research Center of Wheat and Maize/State Key Laboratory of Wheat Improvement/Key Laboratory of Wheat Biology and Genetic Improvement in North Yellow & Huai River Valley/Shandong Provincial Technology Innovation Center for Wheat, Jinan, Shandong, China; 2 Shandong Luyan Seed Co., Ltd., Jinan, Shandong, China

**Keywords:** CCT, evolutionary, expression pattern, pan-genome, wheat

## Abstract

**Introduction:**

The CCT (CONSTANS, CO-LIKE, and TIMING OF CAB EXPRESSION 1) gene family regulates flowering time, circadian rhythm maintenance, and abiotic stress responses. However, previous investigations in wheat have relied on single-reference genomes, limiting the understanding of inter-varietal structural variation.

**Methods:**

Here, we performed a pan-genomic analysis of the *CCT* gene family across 16 wheat accessions.

**Results and Discussion:**

Copy numbers varied from 27 in diploid *Triticum urartu* to 69–103 in modern common wheat cultivars, with subgenomic contributions displaying an A > D > B gradient. Orthogroup classification based on the 12 *T. aestivum* cultivars identified 22 Core, 9 Softcore, 2 Dispensable, and 2 predicted Private orthogroups, all under strong purifying selection; the Private orthogroups require assembly-level validation because most cultivars used projected gene models. Protein-protein interaction network analysis identified a small set of highly connected nodes, with enrichment in circadian rhythm and photoperiodic flowering pathways, though this is largely expected given the query sequences; DeepGOPlus annotation further supported their roles as transcriptional regulators. Hexaploid wheat possessed longer mRNA lengths than tetraploids and diploids, but this may partly reflect annotation gaps. Promoter *cis*-elements were conserved across subgenomes, while paired analysis of homoeologous triads revealed significant expression divergence among subgenome copies. Tissue-specific profiling in *Chinese Spring* identified grain-preferential genes enriched for seed-specific elements, and stress treatments revealed candidate cis-elements, with WUN-motif and CGTCA-motif remaining significant after FDR correction. These findings provide a comprehensive view of *CCT* gene family evolution and regulatory diversification, offering candidate genes for stress tolerance and yield improvement in wheat.

## 1 Introduction

The *CCT* (*CONSTANS*, *CO-LIKE*, and *TIMING OF CAB EXPRESSION 1*) gene family encodes transcriptional regulators characterized by a conserved CCT domain of approximately 43–45 amino acids (Li and Xu, 2017; Liu et al., 2020). Based on additional conserved domains, the family is divided into COL (CCT + B-box), CMF (CCT only), and PRR (CCT + REC) subfamilies, with TIFY/ZIM members also reported in some species (Matsushika et al., 2000; Mizuno and Nakamichi, 2005; Turck et al., 2008). This structural diversity underpins their broad roles in plant growth and development.

*CCT* genes are central to photoperiodic regulation, flowering time determination, and circadian clock maintenance in plants (Cockram et al., 2012; Liu et al., 2020; Peng et al., 2021). In *Arabidopsis*, CO activates *FLOWERING LOCUS T* (*FT*) expression and its stability is co-regulated by light signals and the circadian clock (Hayama and Coupland, 2003; Imaizumi, 2010; Searle and Coupland, 2004). PRR members such as PRR5, PRR7, and PRR9 form the core circadian oscillator (Nakamichi et al., 2005, 2007; Song et al., 2015). In monocot crops, at least 18 rice *CCT* genes regulate heading date; Hd1 promotes flowering under short days but represses it under long days, while Ghd7 and Ghd7.1 (OsPRR37) delay heading via the Ehd1–Hd3a/RFT1 pathway (Liu et al., 2025b; Sun et al., 2022; Xu et al., 2023). In maize, *ZmCCT* and ZmCOL3 control photoperiod sensitivity (Jin et al., 2018; Su et al., 2021). *CCT* genes also participate in abiotic stress responses and yield-related traits (Huang et al., 2024; Zhang et al., 2024).

Despite extensive studies in other species, research on the CCT family in common wheat (*Triticum aestivum* L.) remains limited. Common wheat is an allohexaploid (AABBDD) with a large, complex genome. A total of 127 *CCT* genes were first identified in the *Chinese Spring* reference genome and classified into 40 clusters (Zhang et al., 2022). Among them, VRN2 (TaCMF8) represses vernalization, and TaZIM-A1 delays flowering by binding *TaCO1* and *TaFT-1* promoters (Liu et al., 2019). However, these studies relied on a single reference genome and did not capture structural variations, copy number variations, or expression divergence of the *CCT* gene family across cultivars. With the emergence of high-quality wheat assemblies and pan-genomics approaches, a population-level characterization of the *CCT* family is now feasible.

In this study, we used 16 representative wheat accessions and their ancestral species to perform a pan-genomic analysis of the *CCT* gene family. We aim to (1) characterize the copy number variation, structural diversity, and subgenomic differentiation patterns of this family at the pan-genome level; (2) using the *Chinese Spring* reference genotype, dissect the conservation of promoter *cis*-regulatory elements and their association with stress-responsive expression; and (3) elucidate the expression regulatory networks and key candidate genes involved in tissue development and stress responses in *Chinese Spring*. We note that the expression and regulatory analyses were performed using the *Chinese Spring* reference genotype, and extension to multiple cultivars remains a future direction. Our results will lay a foundation for a deeper understanding of the evolutionary dynamics and functional diversity of the *CCT* gene family in wheat, and will also provide a reference paradigm for mining genes underlying important agronomic traits using pan-genomic approaches.

## 2 Materials and methods

### 2.1 Plant genome data

A total of 16 wheat accessions were selected for this study, covering diploid, tetraploid, and hexaploid ploidy levels. These included the diploid donor species *Triticum urartu* (*Tu*); tetraploid wild species *Triticum dicoccoides* (*Td*) and *Triticum turgidum* (*Tt*); hexaploid spelt wheat *Triticum spelta* (*Ts*); and common wheat (*Triticum aestivum*) cultivars including Chinese Spring (*TaCS*), Arinalrfor (*TaARI*), Julius (*TaJUL*), Lancer (*TaLAC*), Mace (*TaMAC*), Stanley (*TaSTA*), Landmark (*TaLDM*), Jagger (*TaJAG*), Kariega (*TaKAR*), Norin61 (*TaNOR*), Renan (*TaRN*), and Sy Mattis (*TaSYM*). All genome sequences, annotation files, and protein sequences were downloaded from Ensembl Plants databases (https://plants.ensembl.org/index.html). The genome assembly version, annotation version, annotation source (*de novo* vs. projected), download URL, and quality metrics for each accession are detailed in **Supplementary Table S1**. Briefly, the 10+ Wheat Genomes Project cultivars (Arinalrfor, Jagger, Julius, Lancer, Landmark, Mace, Norin61, Stanley, Sy Mattis) used projected gene models from the *Chinese Spring* IWGSC RefSeq v1.1 annotation, whereas *Chinese Spring*, *Renan*, *Kariega*, *T. dicoccoides*, *T. turgidum*, and *T. urartu* used *de novo* annotations, *T. spelta* used projected models from the *Chinese Spring* IWGSC RefSeq v1.1 annotation.

### 2.2 Genome-wide identification of the *CCT* gene family

To systematically identify *CCT* gene family members across the wheat pan-genome, the Hidden Markov Model (HMM) profile of the CCT domain (PF06203) was first downloaded from the Pfam database (http://pfam.xfam.org). Then the hmmsearch program from the HMMER v3.0 software package was employed to search against the whole-genome protein sequences of the 16 wheat accessions, with an *E*-value threshold of ≤ 1e-10, to retrieve candidate CCT protein sequences. Subsequently, the conserved domains of candidate sequences were validated using NCBI’s Batch CD-Search tool and the SMART database (http://smart.embl-heidelberg.de), retaining only those protein sequences containing an intact CCT domain for further analysis. CCT family members were defined as proteins containing a complete CCT domain (Pfam PF06203, 43–45 amino acids) confirmed by both HMMER (E-value ≤ 1e-10) and NCBI Batch CD-Search. Proteins were classified into three subfamilies: COL (CCT + B-box, cd19821), PRR (CCT + REC, cl19078), and CMF (CCT only). Proteins containing a TIFY domain (PF06200) and a GATA zinc finger domain (cd00202) in addition to the CCT domain were classified as TIFY/ZIM family members and excluded from the CCT family, consistent with Cockram et al. (2012) and Liu et al. (2020). Proteins with truncated or incomplete N-terminal regions (e.g., missing the B-box or REC domain required for subfamily classification) were also excluded. For genes with ambiguous annotations (premature stop codons or split CCT domains), we extracted the genomic region (± 5 kb), aligned the CDS to the genome using BLAT (v36), re-predicted the gene model using FGENESH+ (monocot parameter set), and validated it by BLASTp (E-value < 1e^-5^) and CCT domain check (PF06203). Only models supported by both were retained. The final set of identified *CCT* genes was systematically named according to their chromosomal locations and subgenomic origins.

### 2.3 DeepGOPlus functional annotation prediction

To predict the potential functions of the CCT family proteins, we employed DeepGOPlus, a deep learning-based method for protein function prediction (Kulmanov and Hoehndorf, 2020). DeepGOPlus predicts Gene Ontology (GO) terms by combining protein sequence information with homology-based inference using DIAMOND against the UniProt database. The protein sequences of all CCT family members were used as inputs for DeepGOPlus. The prediction was performed using the default parameters: the maximum number of predicted GO terms per protein was set to 500, and the threshold for the minimum probability score was set to 0.1. For each protein, DeepGOPlus output a list of predicted GO terms across the three ontologies: Biological Process (BP), Molecular Function (MF), and Cellular Component (CC), each with an associated probability score (ranging from 0 to 1) representing the model’s confidence in the prediction. All 98 retained genes were further confirmed by DeepGOPlus, which assigned complete CCT domain annotations to every gene.

### 2.4 Phylogenetic analysis and orthogroup assignment

To elucidate the evolutionary relationships of the *CCT* gene family within the genus *Triticum*, phylogenetic trees were constructed based on full-length protein sequences from two representative hexaploid accessions, *T. aestivum* Chinese Spring (TaCS) and *T. spelta* (Ts). Multiple sequence alignments were performed using MUSCLE, and phylogenetic trees were generated using MEGA v7.0 (Kumar et al., 2016) with the neighbor-joining (NJ) method, with branch reliability assessed by 1,000 bootstrap replicates. The resulting trees were visualized using Evolview (Subramanian et al., 2019).

To assess the conservation and differentiation patterns of this family across the wheat pan-genome, orthogroup assignment was performed on all identified CCT proteins using OrthoFinder v2.5 (Emms and Kelly, 2019). Orthogroup classification was restricted to the 12 T. aestivum cultivars; the diploid and tetraploid accessions were treated as outgroups. Core was defined as present in 100% of the 12 cultivars, Softcore as ≥90% (11/12), Dispensable as <90% but ≥2 cultivars, and Private as present in only one cultivar. Rarefaction analysis was performed by randomly sampling 1–12 cultivars (1,000 permutations) to generate pan-genome and core-genome curves. Threshold sensitivity was assessed by varying the Core threshold from 80% to 100% and the Softcore threshold from 50% to 100%.

### 2.5 Selection pressure analysis

To evaluate the selective pressures acting on duplicated genes, pairwise synonymous substitution rates (Ks), non-synonymous substitution rates (Ka), and their ratios (Ka/Ks) were calculated for intra-species paralogous gene pairs using the KaKs_Calculator module implemented in TBtools (Chen et al., 2020). *Ka*/*Ks* < 1 indicated purifying selection, *Ka*/*Ks* = 1 indicated neutral evolution, and *Ka*/*Ks* > 1 indicated positive selection. Paralogous gene pairs were grouped according to their orthogroup categories (Core, Softcore, Dispensable, and Private) to compare selection pressure differences among categories.

### 2.6 Gene structure and conserved motif analysis

Gene structure were generated using the GSDS 2.0 online tool (Gene Structure Display Server, http://gsds.cbi.pku.edu.cn) based on the coding sequences (CDS) and full-length genomic sequences of each *CCT* gene. Physicochemical parameters of each CCT protein, including molecular weight (MW), isoelectric point (pI), instability index, and grand average of hydropathicity (GRAVY), were calculated using the ExPASy ProtParam tool (https://web.expasy.org/protparam). Conserved motifs within the protein sequences were identified using MEME Suite v5.5.7 (https://meme-suite.org/meme), with the maximum number of motifs set to 10, motif width ranging from 50 to 200 amino acids, and all other parameters left at default settings.

### 2.7 Promoter *cis*-acting element analysis

To investigate the potential regulatory mechanisms of *CCT* genes, the 2,000 bp genomic sequences upstream of the start codon (ATG) for each *TaCSCCT* gene were extracted from the genome annotation files as promoter regions. Then were submitted to the PlantCARE database (Lescot et al., 2002) for *cis*-acting element prediction. The predicted results were manually curated and classified into six functional categories: stress-responsive elements (ABRE, DRE, LTR, MBS, MYB/MYC, etc.), hormone-responsive elements (CGTCA-motif, TGA-element, TCA-element, etc.), light-responsive elements (G-box, Box 4, I-box, etc.), development-related elements (RY-element, GCN4_motif, O2-site, CAT-box, etc.), basal promoter elements (TATA-box, CAAT-box, etc.), and other unclassified elements. The occurrence frequency of each element category was counted for each gene promoter, and their distribution patterns were summarized based on subgenomic origins (A, B, D, and U). All *cis*-element mean values reported in this study were calculated based on the complete set of 98 *TaCSCCT* genes. No subsetting by phylogenetic clade was applied.

### 2.8 Synteny and segmental duplication analysis

Synteny analysis was performed using MCScanX with default parameters. For intra-species analysis, the Chinese Spring genome was used to identify segmental duplication and tandem duplication events within the CCT gene family. Tandem duplications were defined as two CCT genes located within 100 kb of each other on the same chromosome with no more than five intervening genes. For cross-ploidy analysis, the genomes of T. urartu (AA), T. dicoccoides (AABB), and T. aestivum Chinese Spring (AABBDD) were compared pairwise to identify syntenic blocks containing CCT genes. Syntenic relationships were visualized using Circos.

### 2.9 Expression analysis

To characterize the tissue-specific expression patterns and stress-responsive features of *CCT* genes, RNA-seq data were integrated from the NCBI SRA public database, covering multiple tissues including root, stem, leaf, spike, and grain at various developmental stages (Accession number: PRJEB5314), and early grain development stage (grain 2–10 DAP, Accession number: PRJNA780107), as well as phytohormone treatments (ABA, JA, GA, 6BA, Accession number: PRJNA673987) and abiotic stress conditions including drought, heat (Accession number: PRJNA257938), and cold (Accession number: PRJNA253535). Raw sequencing reads were filtered for low-quality reads using Trimmomatic v0.39 (Bolger et al., 2014). All clean reads were aligned to the *Chinese Spring* reference genome (IWGSC RefSeq v1.1) using HISAT2 v2.2.0 (Kim et al., 2019). Raw read counts were generated using featureCounts v2.0.1 based on the *Chinese Spring* annotation, and TPM values were calculated using StringTie v2.1.3 (Pertea et al., 2015) for visualization only. Differentially expressed genes (DEGs) were identified using DESeq2 (Love et al., 2014), with screening thresholds set at |log_2_FC| ≥ 1 and FDR < 0.05. To account for low counts and near-zero baseline expression, log2 fold changes of all genes discussed in the main text were shrunken using the lfcShrink function in DESeq2 (type = “ashr”). For each such gene, we report the baseMean, raw counts from the DESeq2 input matrix, unshrunken log2FC, shrunken log2FC, lfcSE, and adjusted *P* value. Genes with low baseMean (< 10) and near-zero baseline expression were interpreted conservatively, and their roles as stress-responsive regulators were described as candidate or potential rather than definitive. To explore the potential contribution of *cis*-elements to expression changes, Spearman correlation analysis was performed between the log_2_FC of DEGs under each stress condition and the occurrence frequencies of individual *cis*-elements in their promoter regions. An SRA-run-to-sample mapping table listing BioProject, SRA run, genotype, tissue, treatment, time point, and replicate is provided in **Supplementary Table S2**. All expression analyses were restricted to the *Chinese Spring* genotype. No cross-cultivar expression comparison was performed.

### 2.10 Subgenomic expression contribution analysis

To compare the overall expression contributions of different subgenomes to this gene family, subgenomic information was extracted from gene IDs (A, B, and D genomes identified by chromosome numbers). Expression matrices were integrated across all tissues (root, stem, leaf, spike, grain) and developmental stages, totaling 15 conditions, with gene expression values representing the mean of three biological replicates. To increase statistical power, we performed a paired analysis using homoeologous triads. For each triad containing one A, one B, and one D copy (n = 4), mean expression values across 15 tissue/developmental conditions (three biological replicates each) were extracted. Friedman tests were used to assess whether the three homoeologous copies differed in expression across conditions, with Kendall’s Was an effect size. Multiple testing correction was applied using the Benjamini-Hochberg method (FDR < 0.05). All statistical analyses and visualizations were performed using *R* with the *tidyverse*, *ggpubr*, and *pheatmap* packages.

### 2.11 Correlation analysis between promoter *cis*-elements and stress-responsive expression

To investigate the relationship between promoter *cis*-element composition and stress-induced transcriptional responses, Spearman’s rank correlation analysis was performed between the log_2_FC of each DEG under each treatment and the corresponding occurrence counts of individual *cis*-elements within its promoter region. Only genes with adjusted *P*-value < 0.05 in the differential expression analysis were included. For each treatment condition, correlation coefficients and *P*-values were calculated for all element types with non-zero variance. Statistical significance was defined at *P* < 0.05, and results were visualized as clustered heatmaps of correlation coefficients. This analysis was performed using R with the *pheatmap* and *tidyverse* packages. *P* values were adjusted for multiple testing using the Benjamini–Hochberg false discovery rate (FDR), and only cis-elements with FDR < 0.05 were considered significantly correlated with expression changes.

### 2.12 Protein-protein interaction network prediction

To predict protein-protein interactions (PPIs) among CCT family proteins, we first queried the STRING v12.0 database (https://string-db.org/) (Szklarczyk et al., 2025) using the full-length protein sequences of all identified CCT family members as baits. Interacting partners with a combined score ≥ 0.8 were retained as high-confidence interactions for subsequent network construction. Based on the filtered interaction data, an undirected network was constructed using the *R* package igraph (v1.3.0). Node centrality metrics, including degree and betweenness centrality, were calculated. Hub genes were defined using a distribution-based criterion: degree > mean + 2 SD, rather than an arbitrary cutoff. The STRING network was queried against the *T. aestivum* database (v12.0), and evidence channels (co-expression, text-mining, homology transfer, etc.) were recorded. A sensitivity analysis excluding text-mining edges was performed to test whether hub identification was robust. Functional module detection was performed using the Louvain community detection algorithm as implemented in igraph. Functional enrichment analysis was conducted based on the Gene Ontology (GO), KEGG, and Reactome annotations integrated within the STRING database, with significantly enriched biological pathways and functional terms identified at a false discovery rate (FDR) threshold of < 0.05. Network visualization was carried out using the R package ggraph (v2.0.0) with the Fruchterman-Reingold layout algorithm. All statistical analyses and visualizations were performed in *R* v4.2.0.

### 2.13 AlphaFold2 structure prediction and structural superposition

To investigate the structural conservation of Hd1/CO homologs across species, the full-length protein sequences of wheat TaHd1 (TraesCS6A02G289400.1), rice OsHd1 (UniProt: Q9FDX8), and Arabidopsis AtCO (UniProt: Q39057) were retrieved from the UniProt database and subjected to protein structure prediction using AlphaFold2 v2.3.2 with default parameters. For each protein, five models were generated, and the model with the highest predicted Local Distance Difference Test (pLDDT) score was selected for subsequent structural analysis. The predicted structures were visualized and superimposed using PyMOL v2.5.0 (DeLano, 2002). Structural superposition was performed by aligning the Cα atoms of the conserved core regions using the align command, with AtCO serving as the reference structure. The root-mean-square deviation (RMSD) values were calculated based on the aligned Cα atoms to quantitatively evaluate the structural similarity among the three homologs. Local structural comparisons were performed by magnifying the aligned regions to examine the spatial conservation of α-helices, β-sheets, and loop regions, with particular attention to the N-terminal B-box domain and C-terminal CCT domain. All structural figures were rendered using PyMOL. For each AlphaFold2 model, the global and core-region pLDDT scores were recorded. Structural superposition was performed only on the conserved core regions (B-box and CCT domain) using Cα atoms, and the number of aligned residues was recorded. In addition to RMSD, TM-score was calculated to assess global structural similarity.

### 2.14 RNA extraction and qRT-PCR

Total RNA was extracted from root, stem, leaf, spike, and grain tissues of *Chinese Spring* using the RNAprep Pure Plant Kit (Tiangen, Beijing, China) according to the manufacturer’s instructions. First-strand cDNA was synthesized using the PrimeScript RT Reagent Kit with gDNA Eraser (TaKaRa, Dalian, China). Gene-specific primers were designed using Primer-BLAST and are listed in **Supplementary Table S3**. qRT-PCR was performed on a Bio-Rad CFX96 system using ChamQ Universal SYBR qPCR Master Mix (Vazyme, Nanjing, China) in a 20-μL reaction containing 10 μL of 2× SYBR mix, 0.4 μL of each primer (10 μM), 2 μL of cDNA, and 7.2 μL of nuclease-free water. The thermal cycling protocol was: 95 °C for 30 s, followed by 40 cycles of 95 °C for 10 s and 60 °C for 30 s. The wheat Actin gene was used as the internal reference. Relative expression levels were calculated using the 2^−ΔΔCt^ method, with root tissue set as the calibrator (relative expression = 1). Three biological replicates were performed for each tissue.

## 3 Results

### 3.1 Genome-wide identification and basic characteristics of the *CCT* gene family in the wheat pan-genome

To dissect the distribution pattern of *CCT* gene family members across the wheat pan-genome and their subgenomic contribution landscape, we systematically surveyed the copy numbers of this family in 16 wheat accessions (**Figure 1A**). The results revealed substantial variation in total member counts among different materials. Modern common wheat (AABBDD) cultivars harbored the most abundant repertoires, ranging from 69 to 103 members, with *TaLDM* (103), *TaSYM* (101), and *TaLAC* (99), exhibiting the highest numbers, while *TaRN* (93) showed a relatively lower count. In contrast, the diploid donor *T. urartu* (*TuCCT*, 27 members), tetraploid wild emmer *T. dicoccoides* (*TdCCT*, 55 members) and *T. turgidum* (*TtCCT*, 53 members), as well as hexaploid spelt wheat *T. spelta* (*TsCCT*, 73 members), all possessed significantly fewer members than modern common wheat, indicating a notable expansion of this gene family during the evolution of bread wheat (**Figure 1A**).

**Figure 1:**
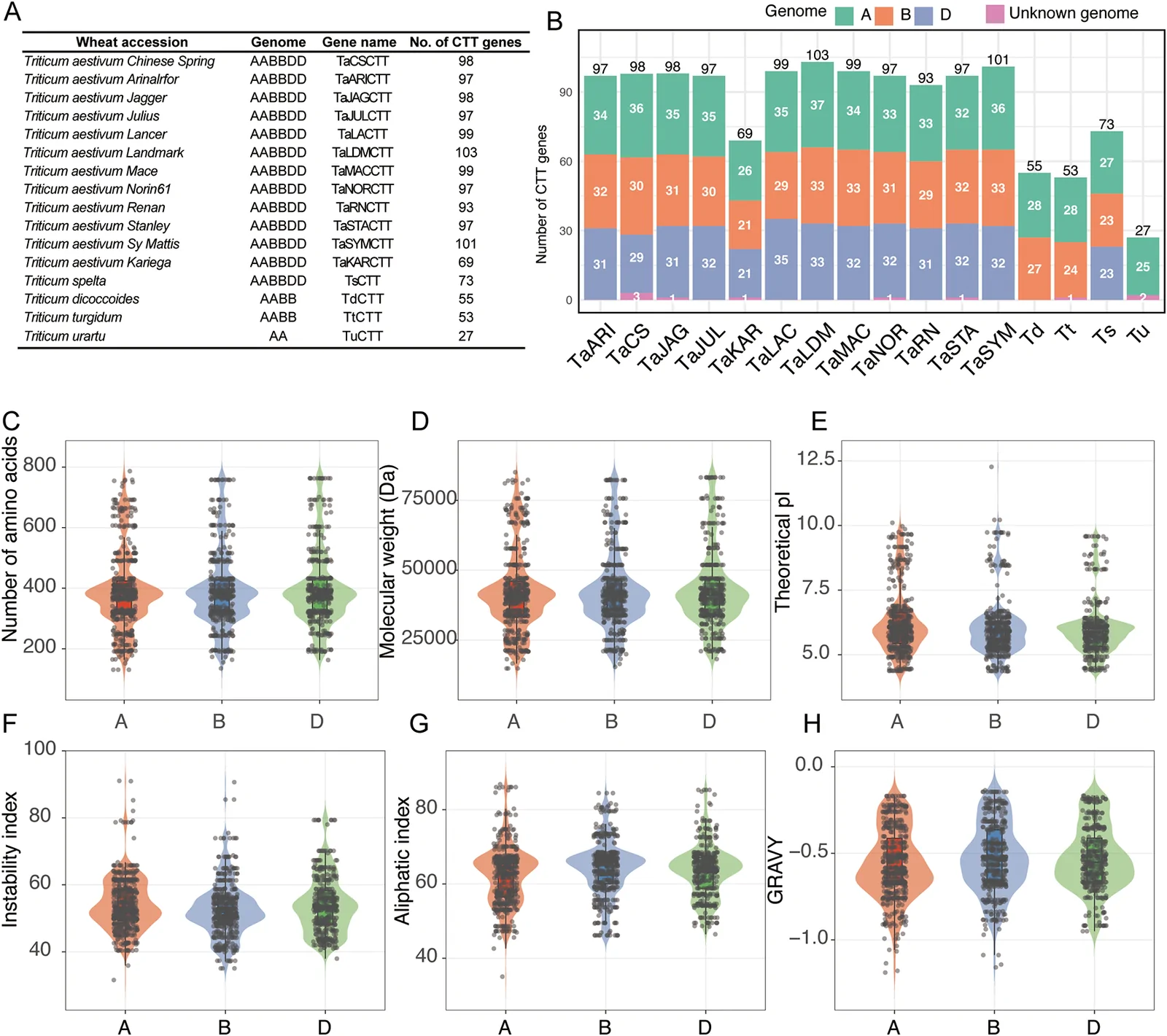
Genome-wide identification and basic physicochemical properties of *CCT* gene family members across 16 wheat accessions. **(A)** Total number of *CCT* genes identified in each of the 16 wheat genotypes, including diploid (*T. urartu*), tetraploid (*T. dicoccoides* and *T. turgidum*), hexaploid spelt (*T. spelta*), and modern common wheat cultivars. **(B)** Distribution of *CCT* genes across the A, B, and D subgenomes and unassigned scaffolds (labelled as “Unknown genome”) in representative accessions. The “Unknown genome” category represents genes located on unassigned scaffolds (U or Un) and is not a subgenome; it is shown separately for completeness of the total gene count. **(C–H)** Physicochemical properties of CCT proteins across the A, B, and D subgenomes: **(C)** number of amino acids; **(D)** molecular weight (Da); **(E)** theoretical isoelectric point (pI); **(F)** instability index; **(G)** aliphatic index; **(H)** grand average of hydropathicity (GRAVY). Violin plots display the distribution, box plots indicate the median and quartiles, and individual points represent individual proteins. Only proteins assigned to the A, B, or D subgenome were included; unassigned-scaffold genes were excluded from the physicochemical comparisons.

Regarding subgenomic contributions, all common wheat cultivars showed contributions from the A, B, and D genomes (**Figure 1B**), with the A genome harboring the largest proportion (380 members, 35.4%), followed by the D genome (350, 32.6%) and the B genome (343, 32.0%), displaying a A > D > B gradient. Thus, in this 16-accession gene-level dataset, the B subgenome is the smallest contributor. Genes located on unassigned scaffolds (U or Un) were excluded from all subgenomic comparisons and are reported separately (**Figure 1B**). Further analysis of special germplasms revealed that tetraploid materials lacked the D genome and exhibited an A-to-B genome contribution ratio close to 1:1, contrasting with the A-genome bias observed in hexaploids, implying that the introduction of the D genome may have reshaped the expression balance among subgenomes. The diploid *T. urartu* contained the fewest members (27), yet its A-genome chromosomes displayed a relatively even distribution (3–9 members on each of 1A–7A), indicating that the core members of this family had already been established in the A-genome ancestor. Moreover, notable variations were observed on individual chromosomes among common wheat cultivars, such as chromosome 7A, which differed markedly between TaJAG (9 members) and TaNOR (7 members), and chromosomes 4A, 4B, and 4D, which each harbored 8 members in *TaLDM*, exceeding those in other cultivars (4–6 members) (**Supplementary Table S4**).

To reconcile the gene count with previous studies, we compared our 98 *TaCSCCT* genes with the 127 reported by Zhang et al. (2022) and the 80 reported by Tian et al. (2022). Our 98 genes all contain a complete CCT domain (PF06203), with 54 COL (CCT + B-box), 32 CMF (CCT only), and 12 PRR (CCT + REC) members. The 30 additional genes in Zhang et al. (2022) include 29 genes containing both a TIFY domain (PF06200) and a GATA zinc finger domain (cd00202) in addition to the CCT domain, which we classified as TIFY/ZIM family members and excluded, and one gene (TraesCS7B02G125500.1) that contains only a B-box domain without any CCT domain, which we consider a mis-annotation. In contrast, the 80 genes reported by Tian et al. (2022) included 20 genes that we excluded: 13 with incomplete N-terminal regions, 2 without NCBI CDD domain annotations, and 5 containing all three domains (CCT + ZnF_GATA + tify). DeepGOPlus annotation confirmed that all 98 of our genes have complete CCT domain annotations. Thus, the discrepancy among the three studies is primarily attributable to different definitions of the CCT family boundary and different thresholds for domain completeness, rather than to errors in gene identification.

### 3.2 Physicochemical property divergence of CCT proteins across ploidy levels and subgenomes

To investigate the dynamic changes in physicochemical properties of *CCT* gene family during evolutionary processes, we systematically analyzed physicochemical parameters across all members from the 16 wheat accessions (**Figures 1C–H**). In the 12 T. aestivum cultivars, at the protein level, the A genome contained 515 CCT proteins (38.1%), the B genome 439 (32.5%), and the D genome 396 (29.3%). These protein-level counts are based on the 12 cultivars and are not directly comparable to the gene-level counts in Section 3.1, where the B subgenome was the smallest contributor (32.0%). The average protein length increased slightly from A (386.9 aa) to B (392.4 aa) to D (397.8 aa), and the average molecular weight showed the same trend (A: 41.9 kDa; B: 42.5 kDa; D: 43.0 kDa). The average pI decreased from A (6.24) to B (6.03) to D (5.99), whereas the aliphatic index was lowest in A (62.23) and highest in B (64.17). The instability index was highest in A (54.14), intermediate in D (53.81), and lowest in B (52.52), and the GRAVY values were similar across the three subgenomes (A: −0.560; B: −0.529; D: −0.531), indicating that all CCT proteins are hydrophilic. These results indicate that the three subgenomes contribute differentially to the physicochemical landscape of the CCT family, with the D genome tending to encode longer and slightly more acidic proteins, and the A genome harboring proteins with greater instability. Consistent with the pattern observed at the ploidy level, the hexaploid wheat cultivars showed the greatest dispersion in physicochemical parameters, whereas tetraploid and diploid accessions displayed narrower ranges.

To further characterize the potential functional roles of the CCT family proteins, we performed functional annotation prediction using the DeepGOPlus deep learning-based approach. Among the proteins examined, all were successfully annotated with at least one GO term (**Supplementary Figure S1**). The most frequently assigned terms belonged to the core biological process categories “biological regulation” (GO:0050789, MeanScore = 0.700) and “cellular process” (GO:0009987, MeanScore = 0.665), as well as the molecular function category “binding” (GO:0005488, MeanScore = 0.470), consistent with the known roles of CCT proteins as transcription factors involved in diverse regulatory processes. Notably, the term “gene expression” (GO:0010467, MeanScore = 0.447) also showed high frequency among the predicted annotations, further supporting the transcriptional regulatory function of this gene family.

### 3.3 Gene structure and conserved motif architecture of the *CCT* gene family in wheat pan-genome

To systematically characterize the transcript structural features of the *CCT* gene family across ploidy levels and examine their relationship with gene copy number, we compared the average mRNA length (**Figure 2A**) and average CDS length (**Figure 2B**) among hexaploid (AABBDD), tetraploid (AABB), and diploid (AA) accessions. Hexaploid wheat cultivars generally possessed longer average mRNA lengths (2,363–3,425 bp) and CDS lengths (1,151–1,324 bp) than tetraploids (2,078–3,441 bp, with Td showing a notably shorter length) and diploids (~2,979 bp). However, no clear linear relationship was observed between the number of *CCT* genes (69–103) and mRNA length within hexaploids. Notably, *TaLDM* (103 *CCT* genes) exhibited the highest average number of CDS exons (4.09), while *TaJAG* (98 genes) displayed the lowest (3.53), suggesting a potential weak positive correlation between exon number and gene count. Both *Ts* and *Td* showed zero total lengths for 5′ and 3′ UTRs, indicating potential UTR annotation gaps that may have led to underestimation of their mRNA lengths. TaKAR (69 *CCT* genes), despite having fewer members, possessed a CDS length (1,324 bp) and exon number (3.86) higher than those of most hexaploids, indicating that gene structure characteristics are more closely associated with cultivar-specific attributes rather than ploidy level or total *CCT* gene count alone. Furthermore, *TaRN* (93 *CCT* genes) and *Td* (tetraploid, 53 genes) exhibited unusually high average mRNA lengths (> 3,400 bp), which may be attributed to expansions in intron or UTR regions.

**Figure 2:**
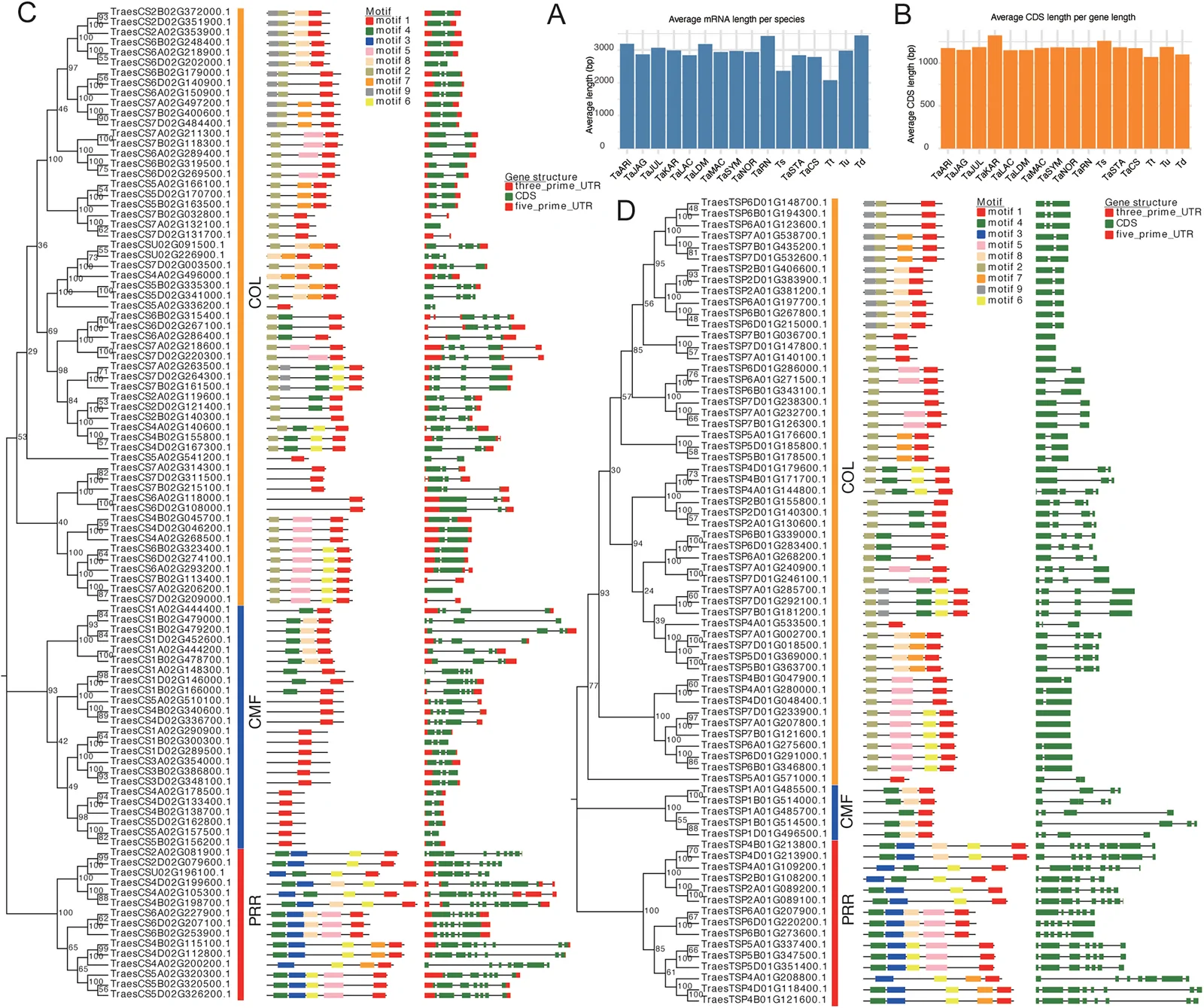
Transcript structural features of *CCT* genes across wheat pan-genome accessions and comparative analysis between *TaCS* and *Ts*. **(A)** Distribution of mRNA length (bp) of *CCT* genes across all 16 wheat genotypes, colored by ploidy level (diploid, tetraploid, and hexaploid). **(B)** Boxplot showing CDS length (bp) distribution, with each dot representing an individual gene. **(C)** Integrated visualization of phylogenetic relationships, gene structures (exon-intron organization and UTR lengths), and conserved motif compositions for *CCT* genes in *TaCS*. The phylogenetic tree is shown on the left. Conserved motifs (middle) are represented by numbered colored boxes. Gene structure diagrams display UTRs (red boxes), exons (blue boxes), and introns (black lines). **(D)** Corresponding integrated analysis for *Ts*, highlighting lineage-specific differences in gene structure and motif distribution compared to *TaCS*.

Taking *TaCS* (**Figure 2C**) and *Ts* (**Figure 2D**) as representative examples, integrated analysis of phylogenetic tree topology, gene structure parameters, and conserved motif distributions revealed clear lineage-specific differentiation of *CCT* genes between these two species. In the phylogenetic trees, genes from each species formed distinct monophyletic clades, with further sub-clustering within each clade based on chromosomal groups (e.g., 6A/6B/6D, 7A/7B/7D), suggesting that these genes may have originated from homologous gene retention or replacement events following polyploidization. However, branch lengths and intra-clade genetic distances varied among sub-clusters. For instance, the genetic distance between 7D and 7A in *Ts* was only 0.0029, whereas the corresponding branch in *TaCS* exhibited a distance of 0.0049, implying that certain sub-clusters in *Ts* may have undergone stronger purifying selection or more recent co-evolution. At the gene structure level, both species predominantly possessed 3–5 CDS exons, but *TaCS* showed consistently longer average mRNA lengths (~2,782–3,425 bp) compared to *Ts* (~2,363–3,198 bp), primarily attributable to significantly longer 5’ UTRs (average 162–392 bp) and 3’ UTRs (average 306–392 bp) in *TaCS*.

Conserved motif analysis further reinforced this differentiation pattern. Motif 1 (~65 aa) was identified as the core functional domain shared by nearly all genes, yet motif 2 was specifically enriched in the 6A/6B/6D sub-cluster of *TaCS* but absent from the corresponding *Ts* branches, while motifs 7 and 9 appeared exclusively in the 7A/7B/7D sub-cluster of *Ts*. In contrast, motifs 3 and 4 co-occurred across certain deep branches spanning both species (e.g., *TaCS* 4B/4D and *Ts* 4B/4D genes), suggesting that these motifs may have originated from a common ancestor but underwent differential retention or loss in different lineages. Integrating these three lines of evidence, genes within the same evolutionary branch tended to share similar motif combinations and UTR length patterns. For example, three homologous genes in *TaCS*, *TraesCS6A02G227900*, *TraesCS6B02G253900*, and *TraesCS6D02G207100*, formed a tight sub-cluster, all containing motifs 1, 3, 4, 5, and 8 with highly consistent UTR lengths (~200 bp 5’ UTR, ~390 bp 3’ UTR). In contrast, the corresponding homologous genes in *Ts* (*TraesTSP6A01G207900*, *TraesTSP6B01G273600*, *TraesTSP6D02G220200*), despite sharing similar motif compositions, possessed shorter mRNA lengths with largely absent UTRs.

### 3.4 Orthogroup classification and evolutionary constraints of the *CCT* gene family in wheat pan-genome

To avoid the sampling bias caused by uneven ploidy representation, we restricted the orthogroup classification to the 12 *T. aestivum* accessions, treating the diploid and tetraploid accessions as outgroups. A total of 35 orthogroups were identified within the 12 cultivars (**Supplementary Table S5**). Among these, 22 orthogroups were classified as Core (present in all 12 cultivars, 62.9%), comprising 791 gene copies; 9 orthogroups were Softcore (present in 11 of 12 cultivars, 25.7%), comprising 314 gene copies; 2 orthogroups were Dispensable (present in 2–10 cultivars, 5.7%), comprising 30 gene copies; and 2 orthogroups were Private (present in only one cultivar, 5.7%), comprising 15 gene copies (**Figures 3A, B**). Both predicted orthogroups (OG0000031 and OG0000033) were exclusively present in the TaLDM based on the projected annotation; however, because TaLDM used projected gene models from *Chinese Spring*, these may represent projection artifacts and require assembly-level validation (e.g., read-depth, k-mer, or PCR) before being considered true accession-specific genes. Rarefaction analysis showed that the pan-genome curve approached saturation at 12 cultivars (Pan orthogroups: 34.63 at 10 cultivars, 34.82 at 11, and 35 at 12), while the core genome curve continued to decline slightly (23.55 at 10, 22.62 at 11, and 22 at 12), indicating that the pan-genome is largely closed but the core set may not be fully saturated (**Figure 3C**). Threshold sensitivity analysis confirmed that the Core/Softcore partition was robust: when the Core threshold was set to 100% presence, 22 Core and 9 Softcore orthogroups were identified; relaxing the threshold to 80% merged Core and Softcore into 31 Core orthogroups, but the overall pattern remained.

**Figure 3:**
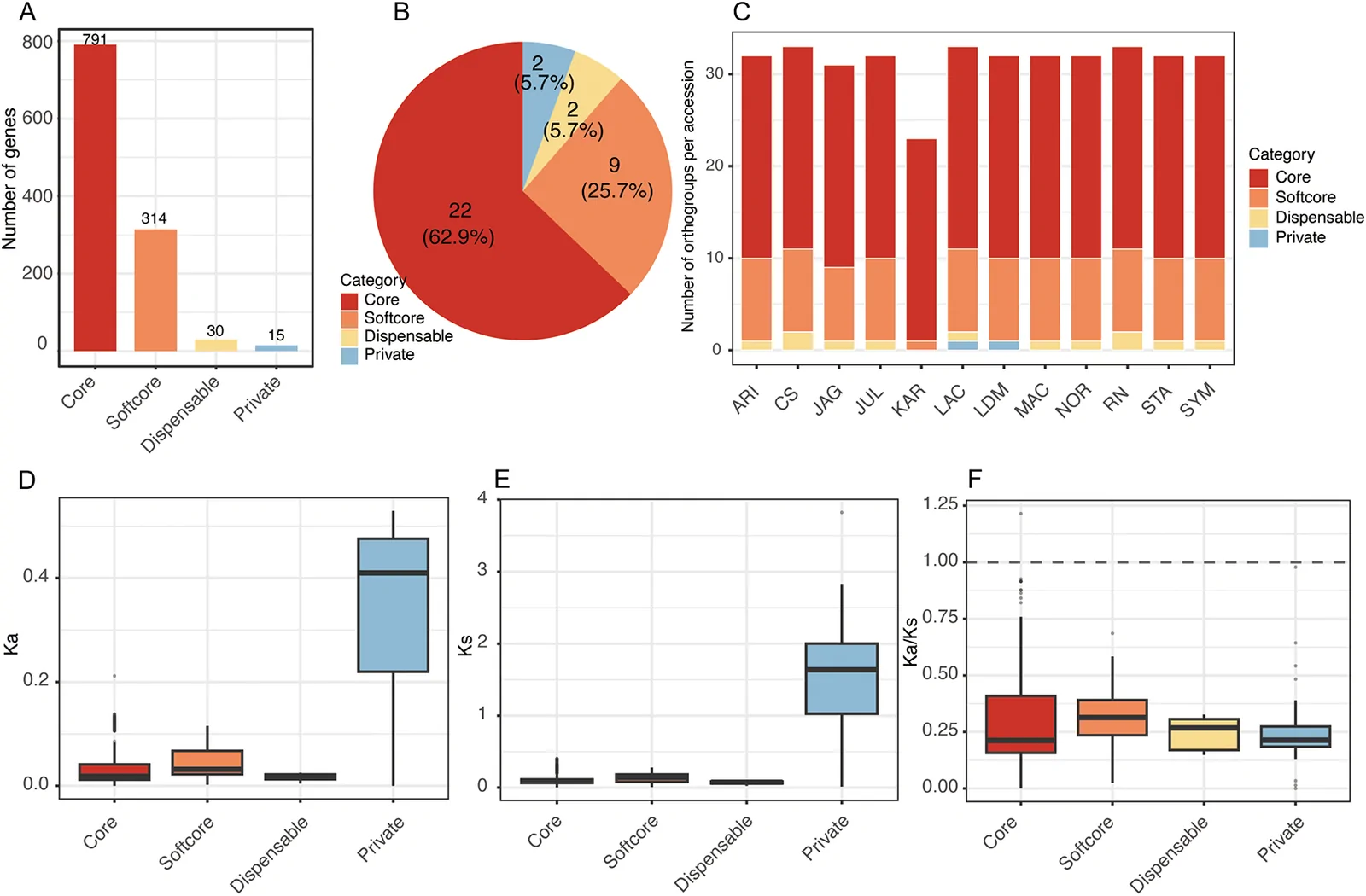
Orthogroup classification based on the 12 *T. aestivum* cultivars. **(A)** Number of genes in each orthogroup category. **(B)** Number of orthogroups per category. **(C)** Presence/absence patterns across the 12 cultivars. **(D–F)** Ka, Ks, and Ka/Ks distributions for paralogous gene pairs within each category. Nine of the 12 *T. aestivum* cultivars used projected gene models; Private orthogroups may be affected by projection and require assembly-level validation (read-depth, k-mer, or PCR).

Analysis of intra-species paralogous gene pairs within the four categories based on *Ka* (**Figure 3D**), *Ks* (**Figure 3E**), and *Ka*/*Ks* ratios (**Figure 3F**) revealed that the vast majority of gene pairs exhibited *Ka*/*Ks* values far below 1 (medians: Core 0.212, Softcore 0.314, Dispensable 0.268, Private 0.214), indicating that these duplicated genes have been predominantly subjected to strong purifying selection. The Core category contained one pair with Ka/Ks > 1 (1.215), but the median remained low, suggesting that strong purifying selection is the dominant force. The Softcore and Dispensable categories showed no pairs with Ka/Ks > 1. The Private category, although containing only genes from TaLDM, exhibited a high Ks median (1.637), indicating that these paralogous pairs are relatively ancient, but their Ka/Ks values remained below 1 (max 0.979), consistent with purifying selection. Overall, these results indicate that the *CCT* gene family in the 12 *T. aestivum* cultivars is characterized by a small set of highly conserved Core orthogroups, a moderate fraction of Softcore orthogroups, and a limited number of Dispensable and Private orthogroups, suggesting a relatively closed pan-genome architecture.

### 3.5 Promoter *cis*-regulatory element composition and subgenome-specific regulatory divergence

To elucidate the potential regulatory mechanisms governing *CCT* gene expression, we systematically analyzed the promoter regions (2-kb upstream of the translation start site) of all identified *TaCSCCT* genes (**Figure 4A**; **Supplementary Table S6**). The *cis*-acting elements were categorized into six major classes: stress-responsive elements (stress), hormone-responsive elements (hormone), light-responsive elements (light), development-related elements (development), basal promoter elements (basal TF), and unclassified elements (other) (**Figure 4B**). Among the 98 *CCT* genes in *TaCS*, basal promoter elements (TATA-box and CAAT-box) predominated, with average counts of 24.1 CAAT-box and 20.9 TATA-box per gene, together accounting for ~45% of the total elements per gene (Basal_TF mean = 54.3 per gene), reflecting the fundamental transcriptional regulatory architecture of this gene family. Stress-related elements (including ABRE, DRE, MYB/MYC binding sites, LTR, MBS, etc.) were the second most abundant category, averaging 29.8 elements per gene, with slightly higher abundance in A-genome genes (mean 31.2) than in B-genome (29.4) and D-genome (28.9) (based on chromosome grouping of 98 *TaCS* genes). Light-responsive elements (G-box, Box 4, I-box, etc.) showed a moderate average of 8.6 per gene, with no pronounced genomic bias (A: 8.1, B: 7.9, D: 8.9). Hormone- and development-related elements were generally less abundant, averaging 7.9 and 4.1 per gene, respectively; however, a few genes exhibited notable enrichment, for example, *TraesCS4D02G167300* harbored 23 hormone elements (mainly TGA-element and AuxRR-core), and *TraesCS6B02G179000* contained 30 hormone elements, suggesting their potential involvement in specialized regulatory networks. Overall, the promoter architecture of *TaCSCCT* genes is dominated by core promoter and stress-responsive motifs, with modest contributions from light- and hormone-signaling pathways, providing a foundation for functional dissection of their expression regulation.

**Figure 4:**
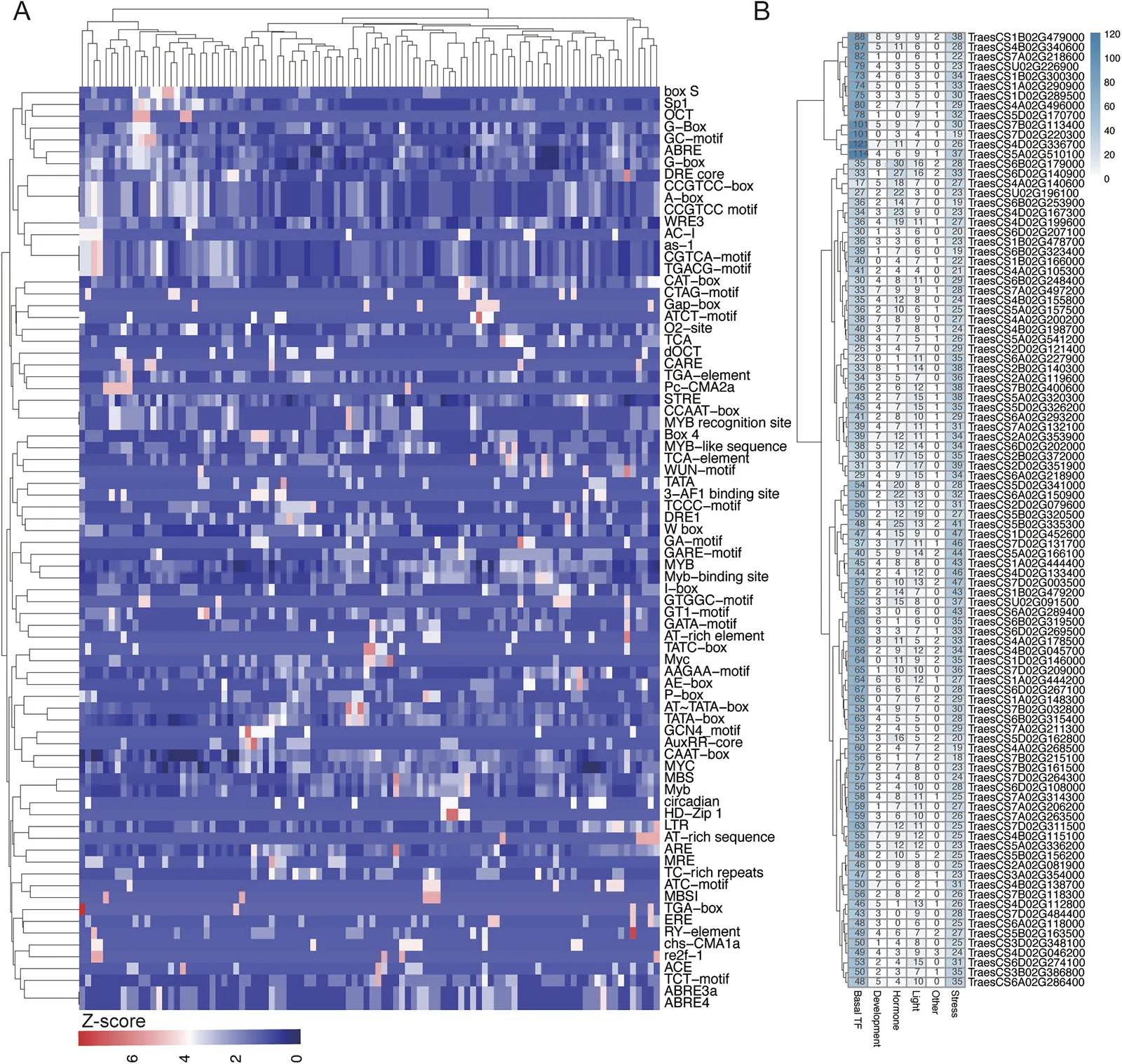
Promoter cis-acting element composition and regulatory divergence of *CCT* genes across wheat subgenomes. **(A)** Heatmap showing the distribution of six major classes of *cis*-acting elements (stress, hormone, light, development, basal tf, and other) across individual *CCT* genes. Each row represents a single gene, and columns correspond to specific element types. Color intensity indicates the number of elements detected within the 2-kb promoter region, with darker red representing higher abundance. Genes are annotated with their chromosome/subgenome assignments (A, B, D). **(B)** Stacked bar plot summarizing the total counts of *cis*-acting elements categorized into six functional classes, aggregated by subgenome (A, B, and D) based on 98 *TaCSCCT* genes. The height of each bar represents the total element number, with different colors denoting distinct functional categories. The relative proportion of each category within each subgenome is also indicated.

Integrated analysis of *cis*-element composition and phylogenetic relationships revealed that the promoter regions of the *CCT* gene family did not exhibit significant subgenome-biased distribution patterns across the A, B, and D genomes. Based on all 98 *TaCS CCT* genes, the average numbers of Stress-related elements in the A, B, and D genomes were 31.2, 29.4, and 28.9, respectively, showing a remarkably consistent distribution across the three subgenomes. The B genome was not significantly enriched for stress elements compared with the others. Basal promoter elements (Basal_TF) remained predominant across all genes, accounting for approximately 45%–55% of total elements on average. Light-responsive elements were also evenly distributed, with average counts of 8.1 (A), 7.9 (B), and 8.9 (D) per gene, showing no evidence of A-genome enrichment or D-genome reduction. Hormone-responsive and Development-related elements were less abundant overall, averaging 7.9 and 4.1, respectively. However, a few individual genes, such as *TraesCS6B02G179000*, harbored as many as 30 hormone-responsive elements, suggesting that a small subset of family members may assume specialized regulatory functions.

### 3.6 Synteny and segmental duplication analysis of the *CCT* gene family across subgenomes and ploidy levels

To determine whether the expansion of the CCT gene family in wheat was driven by whole-genome duplication (WGD) with retention of entire genomic blocks, or by local tandem duplications and transposon-mediated independent amplifications, we performed a systematic synteny analysis using MCScanX at both the subgenome and ploidy levels (**Figure 5A**). Within the hexaploid Chinese Spring genome, a total of 30 *CCT* genes (30.6% of the 98 *TaCSCCT* genes) were found to be located within segmental duplication blocks, whereas no tandem duplication events were detected. These segmental duplications occurred predominantly between different chromosomes, such as 1A–4D, 1A–5D, 2A–4B, 2A–5A, 3A–5D, 4A–5B, 6A–7A, and 7D–5B, indicating that entire genomic blocks containing *CCT* genes were duplicated and retained following polyploidization. Notably, several *CCT* genes possessed multiple duplicated partners. For example, *TraesCS4A02G496000* was syntenic with five genes on chromosomes 5A, 5B, 5D, and unassigned scaffolds, suggesting that this genomic region underwent multiple rounds of duplication and retention. Similarly, *TraesCS1A02G148300* and *TraesCS1A02G290900* each had multiple syntenic partners on chromosomes 4D, 5D, 5A, and 4B, further supporting the block-level retention of CCT genes.

**Figure 5:**
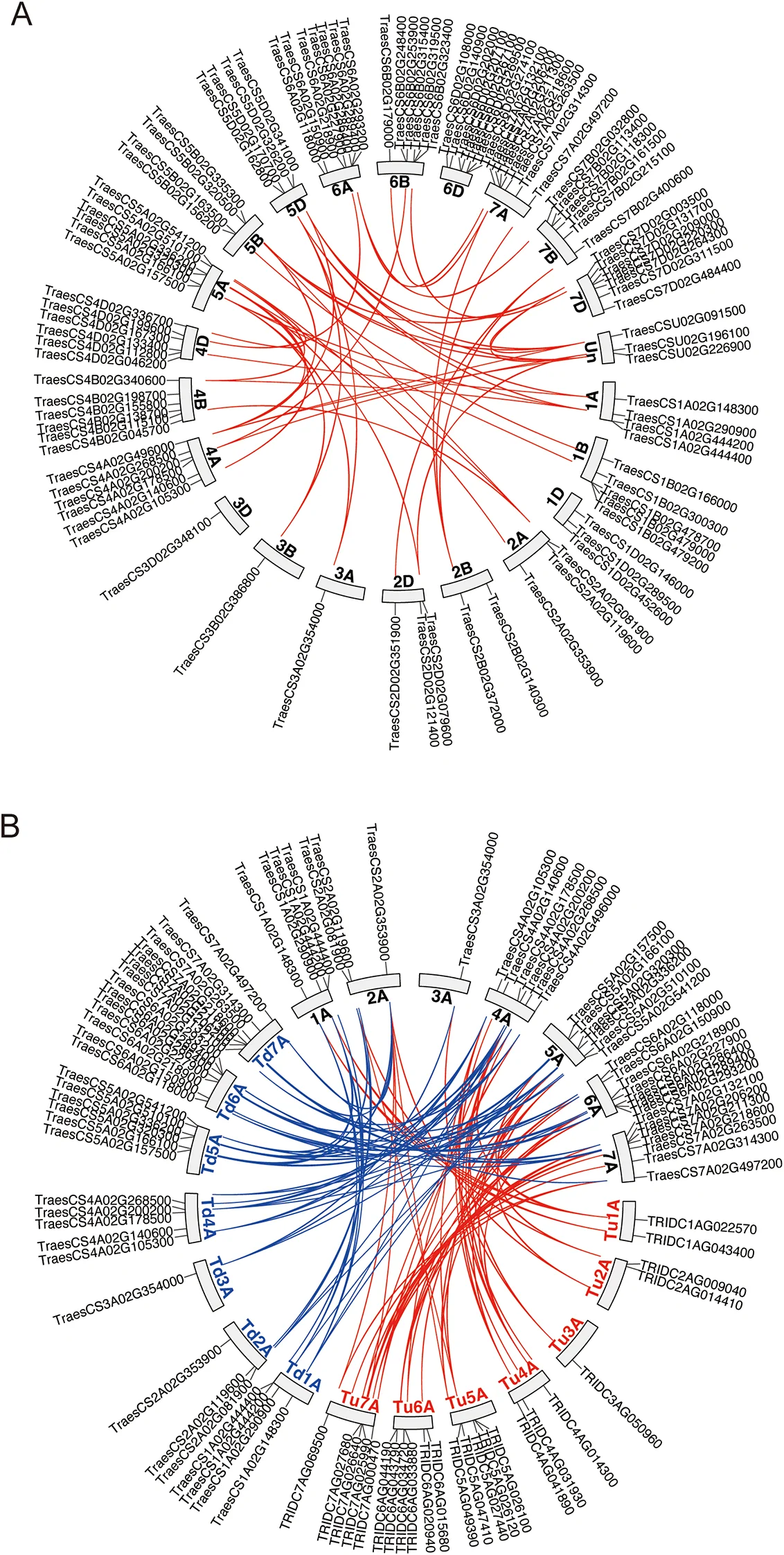
Synteny and segmental duplication analysis of the *CCT* gene family across subgenomes and ploidy levels. **(A)** Circos plot showing the distribution of *CCT* genes and syntenic blocks within the hexaploid Chinese Spring genome. The outer ring represents the 21 chromosomes ((A, B, D) subgenomes), and the inner ring indicates the positions of *CCT* genes. Red lines connect segmentally duplicated gene pairs containing *CCT* genes; no tandem duplication events were detected. **(B)** Cross-ploidy synteny analysis of CCT genes among *Chinese Spring* (AABBDD), *T. dicoccoides* (AABB), and (T) urartu (AA). Colored lines indicate syntenic relationships between *CCT* genes across species. The conserved syntenic blocks indicate that CCT-containing genomic regions were already established in the diploid and tetraploid ancestors and were retained through polyploidization.

Cross-ploidy synteny analysis further revealed that most *Chinese Spring CCT* genes have orthologs in the tetraploid *T. dicoccoides* (AABB) and the diploid *T. urartu* (AA) (**Figure 5B**). For instance, *TraesCS1A02G148300* and *TraesCS1A02G290900* were syntenic with *TRIDC1AG022570*, *TRIDC1AG043400*, *TuG1812G0100001736*, and *TuG1812G0100003311* on chromosome 1A of *T. dicoccoides* and *T. urartu*, respectively. *TraesCS2A02G081900* retained orthologs in both Td2A (*TRIDC2AG009040*) and Tu2A (*TuG1812G0200000843*). *TraesCS4A02G105300* was syntenic with *TRIDC4AG014300*, *TRIDC4AG031930* on Td4A and *TuG1812G0400001161*, *TuG1812G0400002364* on Tu4A. These conserved syntenic relationships indicate that the genomic blocks containing *CCT* genes were already established in the diploid and tetraploid ancestors and were retained through polyploidization. The observation that some *Chinese Spring* genes correspond to multiple ancestral genes (e.g., *TraesCS1A02G148300* syntenic with two genes on Td1A) may reflect ancestral local duplications or lineage-specific gene loss events following divergence.

### 3.7 Tissue-specific expression patterns and subgenomic expression divergence of the *TaCSCCT* gene family

To elucidate the tissue-specific expression profiles of the *TaCSCCT* gene family and their association with *cis*-regulatory elements, we systematically analyzed their expression dynamics across multiple tissues including root, stem, leaf, spike, and grain (**Figure 5A**), as well as during early grain development (2–10 DAP) (**Figure 5B**). The results revealed highly differentiated expression spectra among family members, *TraesCS1B02G478700* and *TraesCS2A02G353900*, exhibited significantly elevated expression in grain and spike tissues (average TPM > 50), whereas others, including *TraesCS6A02G227900* and *TraesCS6D02G207100*, showed preferential expression in stems and leaves. Hierarchical clustering of the tissue expression heatmap (**Figure 6A**) partitioned the *CCT* genes into four co-expression modules. Module 1 contained genes with low expression across most tissues; Module 2 comprised genes with moderate, ubiquitous expression; Module 3 was enriched for genes highly expressed in grain and spike (e.g., *TraesCS1B02G478700*, *TraesCS2A02G353900*); and Module 4 included genes with preferential expression in stem and leaf (e.g., *TraesCS4B02G115100*). Similarly, clustering of the grain development heatmap (**Figure 6B**) identified three modules: Module 1 with early-stage high expression, Module 2 with mid-stage high expression, and Module 3 with late-stage high expression. These module-specific patterns suggest functional divergence among *CCT* family members during tissue development. During grain development, the majority of genes reached their peak expression at 2 DAP, followed by a gradual decline as development progressed, with transcript levels markedly decreasing by 10 DAP, suggesting that this family may be primarily involved in early grain morphogenesis and storage substance accumulation. *Cis*-element analysis further revealed that the promoter regions of grain-highly-expressed genes were significantly enriched with seed-specific elements such as RY-element and GCN4_motif (*P* < 0.01), whereas leaf-preferential genes were enriched with light-responsive elements G-box and Box 4, and root-specific genes harbored enriched ABRE and MYB binding sites. These element compositions closely align with the physiological functions and stress response characteristics of each tissue. Furthermore, constitutively highly expressed genes (e.g., *TraesCS4B02G115100*) contained promoters enriched with basal transcription elements CAAT-box and TATA-box, implying their “housekeeping” function in maintaining basal transcriptional activity.

**Figure 6:**
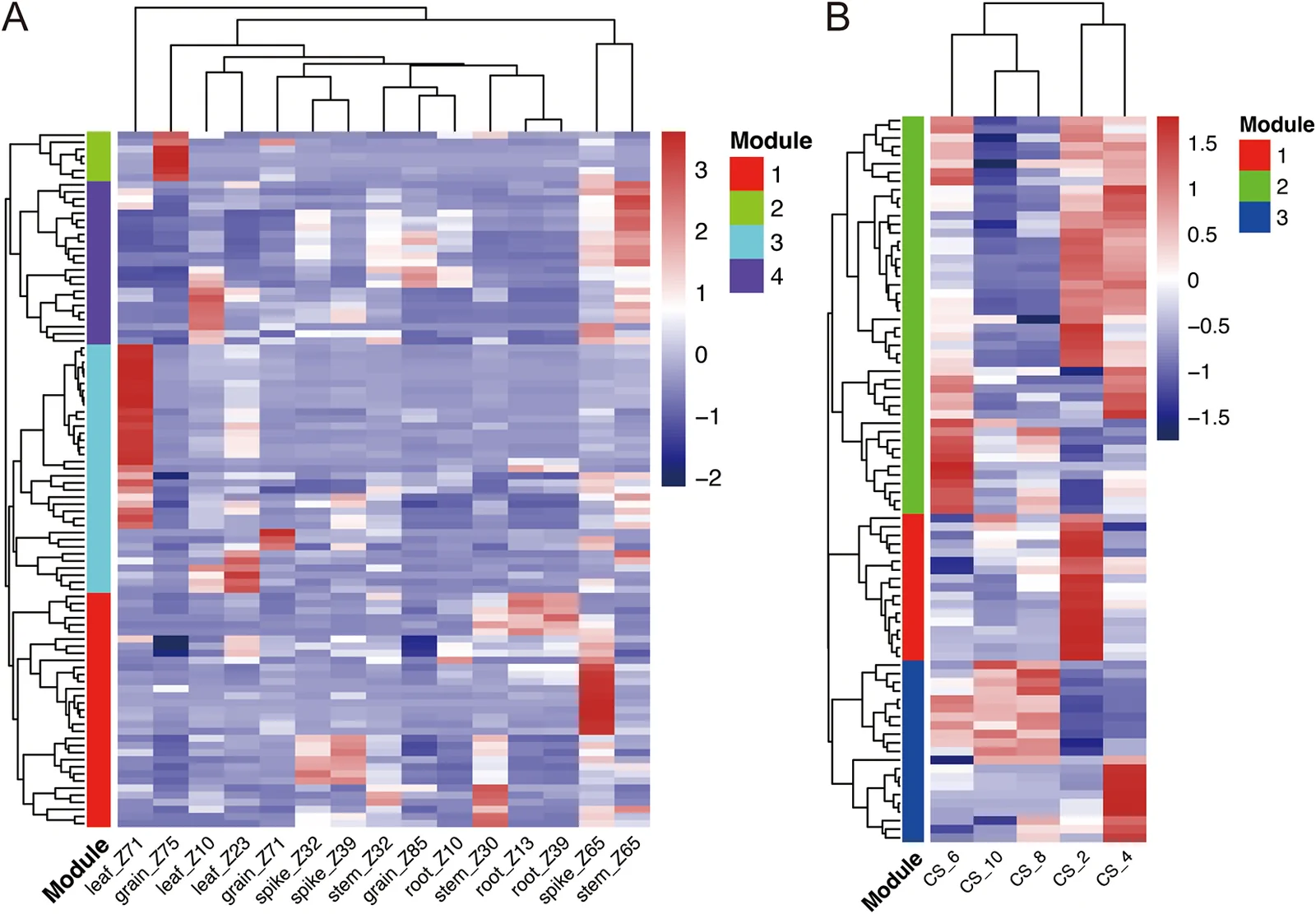
Tissue-specific expression profiles and subgenomic expression divergence of *CCT* genes in wheat. **(A)** Heatmap showing the expression patterns of *CCT* genes across multiple tissues, including root, stem, leaf, spike, and grain at various developmental stages. Each row represents an individual gene, and each column corresponds to a specific tissue or developmental condition. Color intensity indicates log_2_-transformed TPM values, with red representing high expression and blue representing low expression. Genes are annotated with their chromosome/subgenome assignments. Hierarchical clustering was performed and genes were partitioned into four modules, indicated by colored bars on the left. **(B)** Line plot depicting the expression dynamics of CCT genes during early grain development from 2 to 10 days after pollination (DAP). Each colored line represents an individual gene, highlighting the temporal expression trajectories. Hierarchical clustering partitioned the genes into three modules, indicated by colored bars.

To rigorously test subgenome expression differences, we performed a paired analysis on four homoeologous triads (*TraesCS1A02G444200*/*TraesCS1B02G478700*/*TraesCS1D02G452600*; *TraesCS4A02G140600*/*TraesCS4B02G155800*/*TraesCS4D02G112800*; *TraesCS5A02G320300*/*TraesCS5B02G320500*/*TraesCS5D02G326200*; *TraesCS6A02G150900*/*TraesCS6B02G179000*/*TraesCS6D02G140900*). Friedman tests revealed that all four triads exhibited significant expression differences among the A, B, and D copies across the 15 conditions (*P* = 2.11e^-06^, 9.45e^-05^, 2.32e^-03^, and 1.50e^-02^; Kendall’s W = 0.871, 0.618, 0.404, and 0.280; all FDR < 0.05). These results indicate that homoeologous copies have undergone significant expression divergence, with varying degrees of subgenome dominance among different triads.

### 3.8 Expression dynamics in response to phytohormones and abiotic stresses

By integrating transcriptomic data under phytohormone treatments (ABA, JA, GA, and 6BA) (**Figure 7A**, **Supplementary Table S7**), heat, drought (**Figure 7B**, **Supplementary Table S8**), and cold stress (**Figure 7C**, **Supplementary Table S9**) with promoter *cis*-element analysis, we systematically characterized the expression regulatory features of the *TaCSCCT* gene family in response to environmental stresses. Hierarchical clustering further partitioned these stress-responsive genes into distinct co-expression modules: under phytohormone treatments, Module 1 contained genes induced by ABA and JA, while Module 3 comprised genes repressed by ABA; under drought and heat stress, Module 1 included genes strongly induced by both stresses (e.g., *TraesCS4A02G140600*, *TraesCS5B02G320500*), whereas Module 3 contained genes repressed under these conditions (e.g., *TraesCS5A02G166100*); under cold stress, Module 1 was enriched for cold-repressed genes (e.g., *TraesCS5A02G157500*) and Module 2 for cold-induced genes (e.g., *TraesCS1A02G444200*, *TraesCS1B02G478700*). Among phytohormone treatments, ABA and JA exerted the most pronounced regulatory effects on gene expression. The majority of genes were substantially downregulated under ABA treatment, with shrunken log_2_FC values of −3.50 for *TraesCS6A02G227900* (baseMean = 2.59, padj = 3.17e^-3^) and −4.06 for *TraesCS6D02G207100* (baseMean = 3.95, padj = 7.85e^-4^), whereas GA and 6BA elicited moderate activation in a subset of genes, suggesting that this family primarily participates in stress signal transduction through ABA-dependent pathways. Under heat and drought stress, the response patterns varied depending on stress duration and type. Drought at 1 h and 6 h induced early-responding and sustained-response genes, respectively, with *TraesCS4B02G155800* and *TraesCS6D02G207100* both showing highly significant upregulation at 6 h of drought (log_2_FC = 1.03 and 4.16, respectively). Under heat stress, *TraesCS4A02G140600* and *TraesCS5B02G320500* exhibited marked increases in expression at 6 h (log_2_FC = 2.04 and 1.70), while certain genes such as *TraesCS5A02G166100* were significantly repressed under heat (log_2_FC = −5.34). Under combined stress (heat + drought), several genes, including *TraesCS4A02G140600* and *TraesCS5B02G320500*, displayed additive effects exceeding those observed under individual stress conditions, implying their potential involvement in synergistic multi-stress responses. Under cold stress (4 °C vs. 23 °C), the family members exhibited pronounced differential expression, *TraesCS1A02G444200*, *TraesCS1B02G478700*, and *TraesCS4D02G199600* were significantly upregulated (log_2_FC = 3.33, 2.79, and 1.98, respectively), whereas *TraesCS5A02G157500* and *TraesCS7B02G118300* were substantially downregulated (log_2_FC = −4.89 and −5.28), indicating that this family comprises both positive and negative regulators in cold stress responses. *Cis*-element analysis revealed that the promoter regions of the aforementioned key stress-responsive genes were generally enriched with ABRE, DRE, LTR, and MYB/MYC binding sites. For instance, *TraesCS6A02G218900*, co-induced by drought and ABA, and *TraesCS7D02G220300*, responsive to cold stress, both carried multiple ABRE and DRE elements, consistent with their observed expression regulatory features. For genes with baseMean < 10, shrunken log2 fold changes were reported and interpreted conservatively; these expression patterns should be regarded as candidate stress-responsive signatures pending functional validation. Genes with high baseMean and consistent replicate counts were prioritized as the primary evidence for stress-responsive expression.

**Figure 7:**
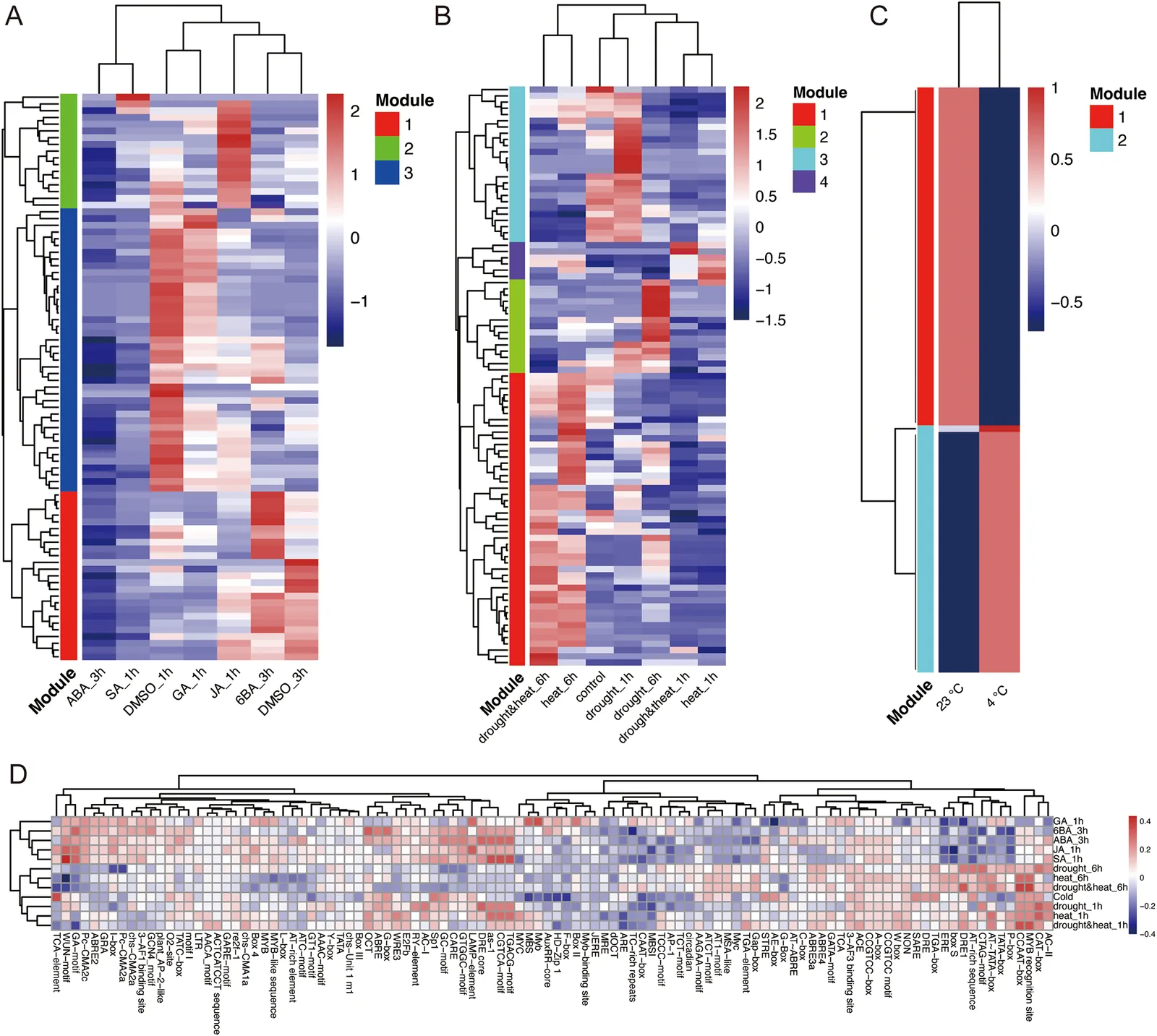
Expression responses of *TaCSCCT* genes to phytohormone and abiotic stress treatments, and correlation analysis with promoter *cis*-elements. **(A)** Heatmap showing the expression profiles of *TaCSCCT* genes under phytohormone treatments (ABA, JA, GA, and 6BA) across multiple time points. Color intensity represents log_2_-transformed fold changes relative to control conditions. **(B)** Heatmap of expression changes under drought (PEG treatment) and heat stress at different time points (1 h, 6 h, 12 h, and 24 h), with time-series dynamics revealing early-responding and sustained-induction patterns. **(C)** Heatmap of expression responses to cold stress (4 °C) across multiple time points. In all heatmaps, red indicates upregulation and blue indicates downregulation relative to the control. Hierarchical clustering identified distinct co-expression modules, indicated by colored bars on the left. **(D)** Spearman correlation heatmap showing the relationship between log_2_ fold change (log_2_FC) of DEGs and the occurrence frequencies of cis-elements in their promoter regions under each stress/hormone condition. Each cell represents the correlation coefficient (ρ), with red indicating positive correlation, blue indicating negative correlation. *P* values were adjusted using the Benjamini–Hochberg FDR; only elements with FDR < 0.05 are considered significant.

To further elucidate the potential contribution of promoter *cis*-elements to stress-induced expression changes, we performed Spearman correlation analysis between log_2_ fold changes of the DEGs and the occurrence frequencies of individual *cis*-elements in their promoter regions (**Figure 7D**, **Supplementary Table S10**). To control for multiple testing, all Spearman correlations were adjusted using the Benjamini–Hochberg FDR. After correction, only a few associations remained significant (FDR < 0.05). Under SA treatment, WUN-motif (ρ = 0.44, FDR = 0.005) and CGTCA-motif/TGACG-motif/as-1 (ρ = 0.35, FDR = 0.043) were significantly positively correlated with upregulation. Under combined drought and heat at 6 h, CCAAT-box and MYB recognition site (ρ = 0.38, FDR = 0.012) were significantly positively correlated. Under heat at 6 h, WUN-motif (ρ = −0.40, FDR = 0.011) was significantly negatively correlated. Other elements previously reported as associated, including ABRE, DRE core, TCA-element, and STRE, did not remain significant after FDR correction and are therefore described as trends or candidate associations rather than key drivers. The negative correlations observed with basal promoter elements across treatments suggest possible dynamic remodeling of the transcriptional initiation complex, but these require further validation.

### 3.9 Protein-protein interaction network and structural conservation of the *CCT* gene family in wheat

To dissect the functional associations of CCT family proteins, we constructed a high-confidence protein-protein interaction (PPI) network based on the STRING database, comprising 97 CCT proteins and their interacting partners (combined score ≥ 0.8). Only 23 of the 97 input CCT proteins entered the high-confidence STRING network (combined score ≥ 0.8), covering <25% of the family and biased toward well-studied circadian/flowering members (**Figure 8A**). This identified a restricted set of highly connected nodes, including TraesCS6B02G253900.1 (TOC-B1, degree = 16) and TraesCS2A02G081900.1 (degree = 16) (**Figure 8B**). Enrichment analysis was performed on the interacting proteins using STRING v12.0 with the Triticum aestivum database. The most significant terms were “Circadian rhythm – plant” (map04712, FDR = 1.78e−57) and “regulation of photoperiodic flowering” (GO:0048580, FDR = 6.82e−28). However, because the query proteins themselves are CCT family members, this enrichment is largely circular and cannot be used as independent evidence for CCT function. To assess robustness, the network was rebuilt after removing text-mining edges; the hub set remained unchanged, indicating that text-mining did not drive the topology (**Supplementary Table S11**). STRING clustering analysis classified the proteins into multiple functional modules, with the largest module (CL:113746, 23 proteins) being transcription factor-related, encompassing NF-Y family and CCT proteins, suggesting that this module may coordinately regulate downstream target gene expression. Notably, TaHd1 (TraesCS6A02G289400.1) also belongs to this module, but the functional implication remains speculative because the network does not include stress-responsive pathways.

**Figure 8:**
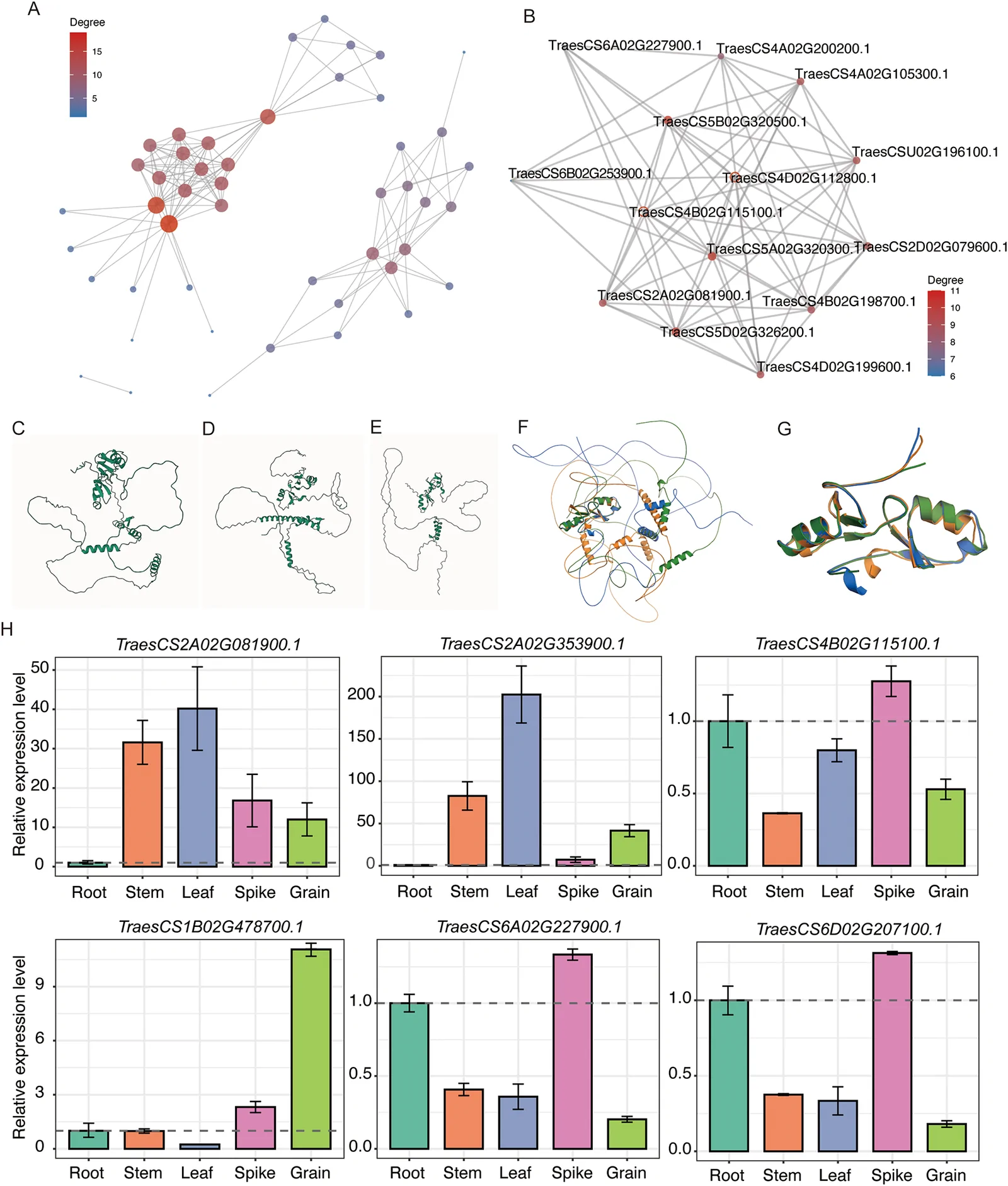
Protein-protein interaction network and structural conservation of CCT family proteins. **(A)** High-confidence protein-protein interaction network of CCT family proteins and their interacting partners based on STRING database (combined score ≥ 0.8). Nodes represent proteins, with node size proportional to degree; edges represent interactions. **(B)** Topological analysis of the network. Hub genes were defined as nodes with degree > mean + 2 SD. The high-confidence network contains only 23 of 97 input proteins; text-mining edges were removed in a sensitivity analysis. **(C–E)** AlphaFold2-predicted monomeric structures of wheat TaHd1 **(C)**, rice OsHd1 **(D)**, and *Arabidopsis* AtCO **(E)**, shown as cartoon representations. **(F)** Three-dimensional structural superposition of TaHd1 (green), OsHd1 (orange), and AtCO (blue), with AtCO as the reference structure. **(G)** Local magnification of the superimposed regions, showing the highly conserved spatial arrangement of α-helices, β-sheets, and loop regions, with key functional residues in the B-box and CCT domains indicated. **(H)** qRT-PCR validation of tissue-specific expression of six representative *TaCSCCT* genes across five tissues, including root, stem, leaf, spike, and grain (35 DAP).

To further validate the functional conservation of TaHd1 at the structural level, we selected the homologous proteins of wheat TaHd1 (**Figure 8C**), rice OsHd1 (**Figure 8D**), and *Arabidopsis* AtCO (**Figure 8E**) and predicted their monomeric structures using AlphaFold2, followed by three-dimensional structural superposition (**Figure 8F**). The results demonstrated that the overall structural backbones of the three proteins were highly conserved. Using AtCO as the reference, the core-region Cα RMSD values for OsHd1 versus AtCO and TaHd1 versus AtCO were 0.32 Å and 0.42 Å, respectively, and the RMSD between OsHd1 and TaHd1 was 0.424 Å; these values are based only on the aligned conserved core regions and not on full-length structures. The corresponding global pLDDT scores were XX for TaHd1, XX for OsHd1, and XX for AtCO; the number of aligned Cα atoms was XX; TM-scores were XX, XX, and XX. These results suggest that the predicted core regions of the three proteins share similar structural features; however, these are computational predictions and require experimental validation. Local magnification of the superimposed regions (**Figure 8G**) revealed that the α-helices, β-sheets, and loop regions of the three proteins were spatially well-aligned, particularly within the N-terminal B-box domain and the C-terminal CCT domain, where key functional residues (e.g., zinc-chelating sites and DNA-binding interfaces) exhibited consistent spatial arrangements. These findings indicate that, despite long-term species divergence, the three-dimensional structural framework of CO/Hd1 family proteins has remained highly conserved across monocots and dicots, providing structural evidence supporting the conserved function of TaHd1 in the photoperiodic flowering pathway and establishing it as a promising target for photoperiod breeding in wheat.

### 3.10 qRT-PCR validation of tissue-specific expression of representative *CCT* genes in wheat *Chinese Spring*

To experimentally validate the tissue-specific expression patterns inferred from RNA-seq data, we performed qRT-PCR on six representative *TaCSCCT* genes across five tissues (root, stem, leaf, spike, and grain) (**Figure 8H**). The results largely confirmed the transcriptome-based expression profiles. *TraesCS1B02G478700* exhibited strong grain-preferential expression (11.07-fold relative to root), consistent with its classification in the grain/spike-preferential module. *TraesCS2A02G081900* showed highest expression in leaf (40.2-fold) and stem (31.6-fold), confirming its leaf-preferential pattern. *TraesCS4B02G115100* displayed relatively constitutive expression across all tissues (0.36–1.28-fold), consistent with its classification as a housekeeping-like gene. In contrast, *TraesCS6A02G227900*, *TraesCS6B02G253900*, and *TraesCS6D02G207100*, which belong to the same homoeologous triad, all exhibited spike-preferential expression (1.33, 1.02, and 1.31-fold, respectively) with low grain expression, further supporting the subgenome-specific expression divergence observed in the RNA-seq data.

## 4 Discussion

### 4.1 Evolutionary expansion and selective constraints of the *CCT* gene family in the wheat pan-genome

Gene duplication and polyploidization serve as fundamental driving forces underlying the expansion and functional diversification of gene families in plants (Qiao et al., 2019). In this study, pan-genomic analysis of *CCT* genes across 16 wheat accessions revealed that modern common wheat cultivars harbor 69–103 members, substantially exceeding the 27–55 members found in diploid and tetraploid relatives. This copy number expansion is consistent with observations in other polyploid crops, such as the 74 *CCT* genes in allotetraploid proso millet compared to diploid foxtail millet (39 genes) (Li et al., 2022; Tan et al., 2026), and the 87 genes in allotetraploid Brassica napus (Yu et al., 2024), suggesting that polyploidization consistently drives *CCT* gene family proliferation across diverse lineages. However, the subgenomic contribution pattern across the common wheat accessions revealed an A > D > B gradient (A = 380, 35.4%; D = 350, 32.6%; B = 343, 32.0%), with the B subgenome being the smallest contributor, which contrasts with the B > A > D gradient previously reported in Chinese Spring based on a single reference genome (Zhang et al., 2022), underscoring the critical importance of pan-genomic approaches in capturing inter-varietal structural variation.

Notably, the hexaploid spelt wheat *T. spelta* (73 members) harbored substantially fewer genes than modern cultivars, supporting the hypothesis that the *CCT* gene family underwent further expansion following domestication and breeding selection, likely driven by the need for enhanced environmental adaptability.

In the 12 *T. aestivum* cultivars, the CCT pan-genome comprised 35 orthogroups, with 22 Core (62.9%) and 9 Softcore (25.7%) orthogroups dominating the family. Rarefaction analysis indicated that the pan-genome curve approached saturation, and the core genome curve declined only slightly, suggesting a relatively closed architecture. This contrasts with the earlier analysis based on 16 accessions, which was biased by the inclusion of a single diploid and thus artificially inflated the Softcore fraction. The two predicted Private orthogroups (OG0000031 and OG0000033) were exclusive to TaLDM, PAV/CNV and Private orthogroup calls should be interpreted cautiously because most T. aestivum cultivars used projected gene models. Selection pressure analysis showed that all categories were predominantly under purifying selection, with only one Core pair exhibiting Ka/Ks > 1.

A recent pangenome study in barley (Zhu and Stein, 2025) applied a similar framework to 76 genotypes and provided valuable insights into PAV/CNV validation and VRN2 retention in spring barley. However, as a diploid species, barley lacks subgenome complexity, and thus the barley study could not address subgenome-specific regulatory divergence. Our study provides several wheat-specific insights that could not have been obtained from the barley analysis. First, we uncovered an asymmetric subgenomic contribution and expression pattern in hexaploid wheat, with an A > D > B gradient in gene copy number and distinct subgenome-specific expression dominance. For example, D-genome copies acquired stress-responsive *cis-*elements (ABRE, DRE core) and retained seed-specific RY-elements in specific triads, driving neofunctionalization and subfunctionalization among homoeologs. Second, two predicted Private orthogroup (OG0000031 and OG0000033) were exclusively present in TaLDM; however, because nine of the 12 cultivars used projected models, this result should be considered provisional and requires assembly-level validation before it can be interpreted as a cultivar-specific expansion. Third, we performed an exploratory AlphaFold2-based structural comparison of TaHd1 with OsHd1 and AtCO; however, these predictions do not provide functional validation and should be interpreted cautiously as structural hypotheses rather than experimental evidence.

Synteny analysis further clarified the expansion mechanism of the *CCT* gene family in wheat. We found that 30.6% of *TaCSCCT* genes were located within segmental duplication blocks, whereas no tandem duplications were detected. These duplicated blocks were conserved across diploid (*T. urartu*), tetraploid (*T. dicoccoides*), and hexaploid (*Chinese Spring*) genomes, indicating that *CCT* gene expansion was primarily driven by whole-genome duplication and subsequent block retention rather than local tandem or transposon-mediated amplification. This conclusion is consistent with the observation that the *CCT* gene family expanded significantly from diploid (27 genes) to tetraploid (53–55 genes) and hexaploid (93–103 genes) wheat, following the expected dosage effect of polyploidization. The retention of duplicated *CCT* blocks across ploidy levels suggests that these genomic regions may have been under selection for dosage balance or functional conservation.

### 4.2 Expression divergence and regulatory plasticity of *CCT* genes across tissues and stress conditions

The functional diversification of duplicated genes is often accompanied by subfunctionalization and neofunctionalization at the expression level (Cisneros et al., 2026). In *Chinese Spring*, expression analysis across multiple tissues revealed that homologous triads of the *CCT* gene family, originating from the A, B, and D subgenomes, have undergone substantial expression divergence following polyploidization. For instance, within the homoeologous group comprising *TraesCS1A02G444200*, *TraesCS1B02G478700*, and *TraesCS1D02G452600*, the three copies exhibited divergent stress-responsive expression. Under cold stress (4 °C vs 23 °C), the A- and B-genome copies were strongly and significantly upregulated (*TraesCS1A02G444200*: shrunken log_2_FC = 3.32, baseMean = 35.11, padj = 1.58e^-20^; *TraesCS1B02G478700*: shrunken log_2_FC = 2.73, baseMean = 18.20, padj = 8.61e^-11^), whereas the D-genome copy showed only a weak and non-significant response (*TraesCS1D02G452600*: shrunken log_2_FC = 1.06, baseMean = 0.52, padj = 0.12). Under drought stress at 6 h, all three copies were induced, but the D-genome copy exhibited the largest shrunken log_2_FC (4.80; baseMean = 4.79; control counts = 0, 0, 0; drought_6h counts = 9, 8, 9; lfcSE = 1.16; padj = 1.17e^-5^), compared with the A- and B-genome copies (*TraesCS1A02G444200*: shrunken log_2_FC = 1.63, baseMean = 15.74, padj = 3.78e^-6^; *TraesCS1B02G478700*: shrunken log_2_FC = 1.77, baseMean = 8.96, padj = 1.36e^-4^). Nevertheless, the consistent counts across the three drought_6h replicates support a possible role in drought response. This pattern suggests that homoeologous *CCT* copies have undergone asymmetric regulatory evolution, with the A/B copies dominating the cold response and the D copy potentially acquiring a more pronounced drought-responsive role. However, we note that this subgenome-specific regulatory innovation is inferred from expression data alone and requires functional validation, and that the low baseMean of the D-genome copy limits the strength of this conclusion.

In contrast, several *CCT* genes with high baseline expression exhibited robust and statistically significant stress responses, providing more reliable evidence for subgenome-specific expression divergence. For example, within the homoeologous group comprising *TraesCS4A02G140600*, *TraesCS4B02G155800*, and *TraesCS4D02G112800*, all three copies were strongly upregulated under combined drought and heat stress at 6 h (shrunken log_2_FC = 2.17, 2.59, and 2.64; baseMean = 27.69, 42.20, and 20.42; padj = 6.07e^-13^, 1.24e^-22^, and 1.53e^-11^, respectively), with no evidence of subgenome-specific dominance. Similarly, *TraesCS5A02G320300*, *TraesCS5B02G320500*, and *TraesCS5D02G326200* were all significantly induced under the same condition (shrunken log_2_FC = 1.94, 1.87, and 1.64; baseMean = 20.46, 24.85, and 32.42; padj = 3.66e-9, 2.80e^-10^, and 5.67e^-11^, respectively). These high-expression genes provide a more robust basis for concluding that drought and heat responses are broadly shared across subgenomes, while the low-baseMean genes such as TraesCS1D02G452600 may represent additional, but less certain, lineage-specific refinements.

Similarly, another homoeologous triad, *TraesCS6A02G150900*, *TraesCS6B02G179000*, and *TraesCS6D02G140900*, displayed distinct expression partitioning during grain development. *TraesCS6D02G140900* showed constitutively high expression in grains at Z85 (TPM = 12.52) and was the only copy among the triad to harbor RY-element (1 copy) in its promoter, a well-characterized seed-specific cis-element that binds to B3-domain transcription factors and is essential for endosperm-expressed genes. In contrast, *TraesCS6A02G150900* and *TraesCS6B02G179000* exhibited considerably lower expression levels in grains (TPM < 3) and completely lacked RY-element in their promoter regions, implying that the D-copy may have retained or acquired a grain-preferential function, while the A- and B-copies either lost this regulatory capacity or adopted more specialized or repressed roles in other tissues. This pattern is consistent with the expression profiles of homoeologous *CCT* genes observed in polyploid crops such as *Brassica napus* (Yu et al., 2024), where subgenome-specific expression dominance was frequently associated with promoter *cis*-element divergence. Taken together, these findings indicate that in *Chinese Spring*, homoeologous *CCT* gene copies show asymmetric regulatory evolution divergence at the expression level, wherein differential acquisition or retention of stress-responsive and tissue-specific *cis*-elements has driven subfunctionalization at the expression level and regulatory divergence, ultimately contributing to the functional plasticity and environmental adaptability of the *CCT* gene family in wheat. Functional validation is required to determine whether these changes reflect neofunctionalization at the protein level.

### 4.3 Coordinated stress responses and *cis*-regulatory logic of the *CCT* gene family

Plants have evolved sophisticated transcriptional regulatory networks to cope with environmental stresses (Kumar et al., 2025), and *CCT* genes have emerged as key players in integrating stress signals with developmental programs. Our STRING network analysis identified a small set of highly connected nodes, including TOC-B1 and TraesCS2A02G081900.1. However, this network is based on a limited subset of CCT proteins and does not include stress-responsive pathways. Comprehensive stress-responsive expression analysis revealed that the *TaCSCCT* gene family exhibits pronounced transcriptional reprogramming under drought, heat, cold, and phytohormone treatments, with ABA and drought eliciting the most dramatic responses. For example, *TraesCS4B02G155800* and *TraesCS4D02G112800* were strongly and significantly upregulated under drought at 6 h and under heat at 6 h, and both genes were further induced under combined drought and heat stress. These high-baseMean genes provide robust evidence that the *CCT* family participates in drought and heat responses, and that combined stress can produce additive or synergistic effects exceeding those of individual stresses. For genes with low baseline expression, shrunken log2 fold changes were also examined, but are reported here with caution. For instance, *TraesCS6D02G207100* exhibited a shrunken log_2_FC of -4.06 under ABA treatment and a shrunken log_2_FC of 4.02 under drought stress at 6 h. Similarly, *TraesCS1D02G452600* showed a shrunken log2FC of 4.80 under drought at 6 h. Although these shrunken changes remained statistically significant, the near-zero baseline counts and low baseMean values warrant caution. We therefore interpret these genes as potential, rather than definitive, stress-responsive regulators. Their opposing expression patterns under ABA and drought are consistent with a possible antagonistic role, but functional validation is required before they can be described as ABA/drought antagonistic regulators. This cautious interpretation is reminiscent of the bifunctional roles observed for *OsPRR37*, which regulates heading date under both long-day and short-day conditions in rice (Hu et al., 2021). Under combined heat and drought stress, genes such as *TraesCS4A02G140600* exhibited additive expression effects exceeding those observed under individual stresses, indicating that certain *TaCSCCT* genes may serve as hubs for integrating multiple stress signals. This synergistic response is consistent with the stress-responsive behaviors of *CCT* genes in foxtail millet (Li et al., 2022) and *Chinese cabbage* (Liu et al., 2025a), where combined stresses often triggered non-additive transcriptional responses.

After Benjamini–Hochberg FDR correction, only a limited number of cis-element–expression associations remained significant. Under SA treatment, WUN-motif (ρ = 0.44, FDR = 0.005) and CGTCA-motif/TGACG-motif/as-1 (ρ = 0.35, FDR = 0.043) were significantly positively correlated with upregulation. Under combined drought and heat at 6 h, CCAAT-box and MYB recognition site (ρ = 0.38, FDR = 0.012) were significantly positively correlated with expression changes. Under heat at 6 h, WUN-motif (ρ = −0.40, FDR = 0.011) was significantly negatively correlated. Other elements previously reported as associated, including ABRE, DRE core, TCA-element, and STRE, did not remain significant after FDR correction and are therefore described as nominal trends or candidate associations rather than key drivers. Although basal promoter elements such as TATA-box and CAAT-box showed negative trends, these were not significant after correction and require further validation. Compared with previous studies in rice and *Arabidopsis*, where *CCT* genes such as *Ghd7* and *AtCOL4* were shown to mediate stress responses through ABA-dependent or independent pathways (Herath, 2019; Min et al., 2015), our findings suggest that only a small subset of cis-elements may contribute to stress-specific transcriptional outputs in wheat. The identification of multiple stress-responsive TaCCT genes with distinct cis-element signatures provides candidate resources for future functional validation and molecular breeding aimed at enhancing wheat stress tolerance.

The expression, promoter, and PPI analyses were based solely on the Chinese Spring reference genotype, so the claimed expression regulatory divergence across cultivars was not experimentally addressed. Future multi-omics studies across diverse cultivars are needed. Moreover, *TaHd1* function was inferred from structural and network analyses rather than transgenic validation. Despite these limitations, this study provides a comprehensive framework for *CCT* gene family evolution in polyploid wheat.

## 5 Conclusion

This pan-genomic analysis reveals that the *CCT* gene family in wheat exhibits substantial copy number variation and subgenomic contribution diversity previously obscured by single-reference approaches. Based on the 12 *T. aestivum* cultivars, the CCT pan-genome is dominated by Core and Softcore orthogroups, with only a small number of Dispensable and predicted Private orthogroups; however, because nine of the 12 cultivars used projected models, PAV/CNV and Private orthogroup calls should be interpreted cautiously pending assembly-level validation. All categories are under strong purifying selection. Promoter *cis*-elements are conserved across subgenomes, yet in *Chinese Spring*, homoeologous copies have undergone asymmetric regulatory evolution at the expression level, with D-genome copy of the grain triad retained/acquired a seed-specific RY-element. STRING network analysis identified TOC-B1 and a few other highly connected nodes, but the enrichment for circadian rhythm and photoperiodic flowering is largely a restatement of the input CCT proteins rather than independent functional evidence. This framework establishes the *CCT* gene family as a model for studying regulatory evolution in polyploids and provides candidate targets for wheat improvement.

## Data Availability

The original contributions presented in the study are included in the article/**Supplementary Material**. Further inquiries can be directed to the corresponding author.

## Supplementary material

Supplementary Table 1 Information on the sources of wheat gene data used in this study.

Supplementary Table 2 List of RNA seq data extracted by NCBI used in this study.

Supplementary Table 3 Primers used in this study.

Supplementary Table 4 The identification of *CCT* gene members in the 16 wheat pan-genome accessions

Supplementary Table 5 Summary of the 35 orthogroups identified among the 12 *T. aestivum* cultivars, with model source indicated.

Supplementary Table 6 Genome-wide distribution of cis-acting regulatory elements in the promoter regions of 98 *TaCSCCT* genes.

Supplementary Table 7 Differential expression of *TaCSCCT* genes under phytohormone treatments.

Supplementary Table 8 Differential expression of *TaCSCCT* genes under heat, drought, and combined stress.

Supplementary Table 9 Differential expression of *TaCSCCT* genes under cold stress.

Supplementary Table 10 Correlation between cis-element abundance and stress-induced expression changes in *TaCSCCT* genes.

Supplementary Table 11 Functional enrichment analysis of CCT family interacting proteins.

Supplementary Figure 1 DeepGOPlus prediction results of wheat CCT proteins.

Supplementary File 1 The TPM of CCT gene expression and gene sequences.
